# DPYSL5 is highly expressed in treatment-induced neuroendocrine prostate cancer and promotes lineage plasticity via EZH2/PRC2

**DOI:** 10.1038/s42003-023-05741-x

**Published:** 2024-01-18

**Authors:** Roosa Kaarijärvi, Heidi Kaljunen, Lucia Nappi, Ladan Fazli, Sonia H. Y. Kung, Jaana M. Hartikainen, Ville Paakinaho, Janne Capra, Kirsi Rilla, Marjo Malinen, Petri I. Mäkinen, Seppo Ylä-Herttuala, Amina Zoubeidi, Yuzhuo Wang, Martin E. Gleave, Mikko Hiltunen, Kirsi Ketola

**Affiliations:** 1 Institute of Biomedicine, University of Eastern Finland, Kuopio, Finland; 2 The Vancouver Prostate Centre and Department of Urologic Sciences, University of British Columbia, Vancouver, BC Canada; 3 Institute of Clinical Medicine, Clinical Pathology and Forensic Medicine, University of Eastern Finland, Kuopio, Finland; 4 Department of Environmental and Biological Sciences, University of Eastern Finland, Joensuu, Finland; 5 A.I. Virtanen Institute, University of Eastern Finland, Kuopio, Finland; 6 BC Cancer Research Centre, Vancouver, BC Canada

**Keywords:** Super-resolution microscopy, Prostate cancer

## Abstract

Treatment-induced neuroendocrine prostate cancer (t-NEPC) is a lethal subtype of castration-resistant prostate cancer resistant to androgen receptor (AR) inhibitors. Our study unveils that AR suppresses the neuronal development protein dihydropyrimidinase-related protein 5 (DPYSL5), providing a mechanism for neuroendocrine transformation under androgen deprivation therapy. Our unique CRPC-NEPC cohort, comprising 135 patient tumor samples, including 55 t-NEPC patient samples, exhibits a high expression of DPYSL5 in t-NEPC patient tumors. DPYSL5 correlates with neuroendocrine-related markers and inversely with AR and PSA. DPYSL5 overexpression in prostate cancer cells induces a neuron-like phenotype, enhances invasion, proliferation, and upregulates stemness and neuroendocrine-related markers. Mechanistically, DPYSL5 promotes prostate cancer cell plasticity via EZH2-mediated PRC2 activation. Depletion of DPYSL5 decreases proliferation, induces G1 phase cell cycle arrest, reverses neuroendocrine phenotype, and upregulates luminal genes. In conclusion, DPYSL5 plays a critical role in regulating prostate cancer cell plasticity, and we propose the AR/DPYSL5/EZH2/PRC2 axis as a driver of t-NEPC progression.

## Abstract (web summary)

Androgen receptor (AR) suppresses DPYSL5, and overexpression of DPYSL5 promotes prostate cancer cell plasticity via EZH2-mediated PRC2 activation, suggesting that the AR/DPYSL5/EZH2/PRC2 axis may act as a driver of treatment-induced neuroendocrine prostate cancer.

## Introduction

Since the introduction of second-generation androgen receptor (AR) targeting medication for castration resistant prostate cancer (CRPC), the prevalence of lineage plasticity and histologic transformation to highly aggressive treatment-induced neuroendocrine prostate cancer (t-NEPC) has increased^1^. Neuroendocrine transdifferentiation has been detected in 79% of patients with metastatic CRPC, and nearly one in five metastatic CRPC patients develops t-NEPC^1^. The life expectancy after NEPC diagnosis is, on average, 7 months, and treatment options are limited to platinum-based chemotherapy^2,3^. The biological mechanisms of prostate cancer progression to NEPC, particularly how it emerges during the pressure of androgen deprivation therapy (ADT) and what causes treatment resistance, are poorly understood. Moreover, novel therapeutic targets and treatment strategies are desperately needed to synergize with ADT, target this lineage plasticity process, and prevent the treatment-resistant and rapidly progressing lethal form of prostate cancer.

The development of t-NEPC is facilitated by the neuroendocrine (NE) lineage plasticity, involving the transdifferentiation of luminal epithelial prostate cancer cells into NE cells under ADT^1,4,5^. Through this process, these cells acquire similar properties to pre-existing neuroendocrine cells^6^. Specifically, both transdifferentiated prostate cancer cells and de novo neuroendocrine prostate cancer cells exhibit the absence of AR and prostate-specific antigen (PSA). They often express neuronal lineage markers chromogranin A (CGA), neuron-specific enolase (NSE), or synaptophysin (SYP). Genetic factors linked to high-risk prostate cancer and the development of NEPC include the loss of tumor suppressors RB1 and TP53, amplification of cell-cycle regulator Aurora Kinase A and MYCN, splicing of transcriptional repressor REST by SRRM4, increased expression of PEG10, and neural transcription factors, as well as prostate cancer cell lineage plasticity inducers BRN2 and ASCL1^5,7–16^. Additionally, Enhancer of zeste homolog 2 (EZH2), the enzymatic core subunit of polycomb repressor complex 2 (PRC2), has been implicated in driving t-NEPC development by activating stemness lineage programming^6,16,17^. EZH2 has been shown to have both PRC2-dependent and independent functions in prostate cancer^18,19^. Several factors promoting EZH2 activation in prostate cancer have been reported, including the upregulation of N-Myc, ASCL1 and SOX2^16,20,21^. Despite the increased knowledge regarding the role of cellular plasticity in t-NEPC progression, understanding the underlying mechanisms of t-NEPC progression under ADT and identifying novel targets to prevent the cellular plasticity-induced luminal-NE lineage switch are still required.

Here, we demonstrate a significant upregulation of neuronal development protein dihydropyrimidinase-related protein 5 (DPYSL5, also known as collapsing response mediator protein 5, CRMP5) in AR-negative treatment-induced neuroendocrine patient and patient-derived xenograft (PDX) tumors. Conversely, no significant expression of DPYSL5 is observed in AR-positive adenocarcinoma or castration resistant patient or the corresponding PDX tumors. DPYSL5 belongs to the family of collapsin response mediator proteins, playing a role in axon guidance and neurite outgrowth during neural development and regeneration^22,23^. DPYSL5 itself promotes neurite outgrowth and neurite branching in hippocampal neurons^24^, and its expression is required for normal development of Purkinje neurons^25^. Previous studies have shown that DPYSL5 mediates its effects on neurite outgrowth through interaction with the actin cytoskeleton in growth cones. In addition, DPYSL5 has been implicated in various cancers, with documented upregulation in high-grade lung neuroendocrine carcinoma^26^, colorectal cancer^27^, and in glioblastoma^28^. However, as of now, the involvement of DPYSL5 in prostate cancer remains unexplored.

We have recently discussed the molecular and functional links between neurodevelopmental processes and treatment-induced neuroendocrine plasticity in prostate cancer progression^29^. Given that DPYSL5 is a regulator of neural development and regeneration, we here explored its potential role in prostate cancer antiandrogen resistance and t-NEPC plasticity. To this end, we analyzed DPYSL5 expression in patient samples using our unique CRPC-NEPC cohort, which includes 135 prostate cancer patient samples, encompassing 55 t-NEPC patient samples. The DPYSL5 protein staining intensity significantly correlated with SYP, CGA and NCAM in prostate cancer patients and showed an inverse correlation with AR and PSA. On molecular level, we demonstrated that DPYSL5 expression is under AR regulation, and the NE phenotype is inducible with the clinically used second -generation AR inhibitor Enzalutamide (ENZ) in prostate cancer cells. Moreover, DPYSL5 overexpression increased the expression of neuronal lineage markers and induced neuron-like morphology in prostate cancer cells and chick chorioallantoic membrane (CAM) tumors. DPYSL5 overexpression also led to an upregulation of EZH2 protein levels and increased H3K27 trimethylation, suggesting an activation of the PRC2 complex. In contrast, DPYSL5 depletion in highly differentiated ENZ-resistant cells resulted in decreased EZH2 and truncated JARID2 protein levels, increased expression of luminal markers, and the loss of the neuroendocrine morphology. Furthermore, DPYSL5 depletion potentiated the ENZ-reduced cell proliferation and cell cycle arrest in G1 phase.

Collectively, these findings identify the role of ectopic DPYSL5 driven by ADT as a new molecular mechanism contributing to treatment resistance, neuroendocrine lineage plasticity and the development of t-NEPC.

## Results

### DPYSL5 is highly expressed in t-NEPC patient and NEPC patient derived xenograft tumors

**Fig. 1:**
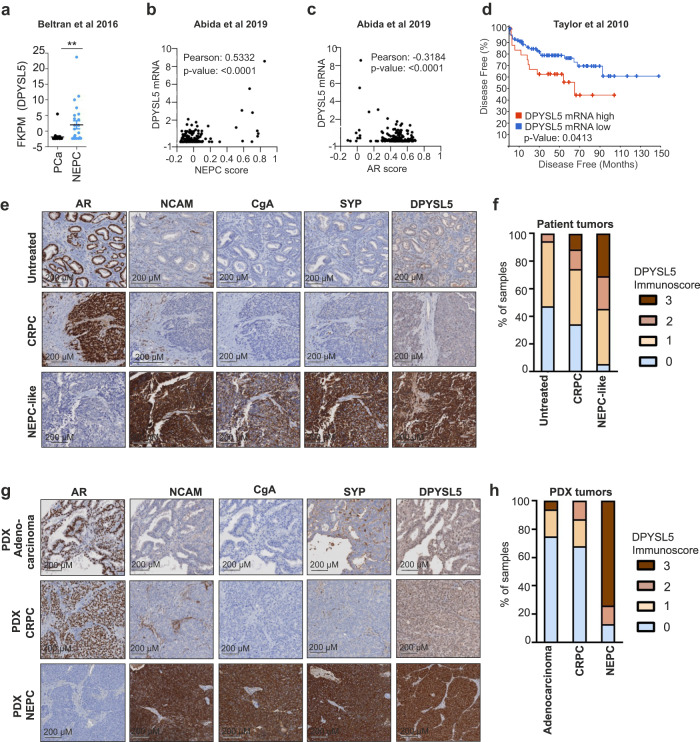
High DPYSL5 expression correlates with increased aggressiveness in prostate cancer. **a** DPYSL5 is highly expressed in NEPC patient tumors in the Beltran et al. 2016 dataset. **b** *DPYSL5* expression shows a positive correlation with NEPC score, while (**c**) a negative correlation with AR Score in the Abida et al. 2019 dataset is observed. **d** High *DPYSL5* mRNA expression correlates with shorter disease-free survival in Taylor et al. 2010 prostate cancer dataset. **e** Intense DPYSL5 staining is observed in treatment-induced NEPC-like patient tissue samples from the Vancouver CRPC-NEPC TMA cohort. To identify tumor areas, H&E slides of retrieved FFPE tissue blocks were reviewed and annotated by pathologists. Tissue cores from donor FFPE blocks (representative of tumor or non-malignant areas, diameter = 1 mm) were assembled in duplicates into a recipient paraffin block with the semi-automated tissue arrayer TMArrayer (Pathology Devices). **f** DPYSL5 staining intensity score is significantly increased in NEPC patient tissue samples (*n* = 55) when compared to untreated (*n* = 37) and CRPC/TURP (*n* = 43) tissue samples. **g** Comparison of DPYSL5 expression intensity patient derived tumor samples (patient derived xenographs, PDX). Samples from patient derived adenocarcinoma (PDX-Adenocarcinoma), CRPC (PDX-CRPC) and NEPC tumors (PDX-NEPC) were included in the analysis. In addition to DPYSL5, staining intensities of AR and NE markers NCAM, CGA and SYP were analyzed. **h** DPYSL5 staining in PDX tumor samples has a significantly high score in NEPC when compared to other prostate cancer tumor types (adenocarcinoma *n* = 16, CRPC *n* = 8 and NEPC *n* = 4).

To investigate DPYSL5 expression in prostate cancer tumors, we examined RNA-seq data from various prostate adenocarcinoma patient cohorts, including tumors with neuroendocrine differentiation. First, we analyzed *DPYSL5* mRNA expression in the NEPC patient cohort from  Beltran et al. 2016^5^ and identified exclusively high *DPYSL5* mRNA levels in clinical NEPC samples compared to prostate adenocarcinoma (Fig. 1a). Next, we utilized the dataset from Abida et al. 2019^30^ to determine if DPYSL5 correlates with NEPC or AR score. Our analysis revealed a positive Pearson correlation between DPYSL5 and higher NEPC score (Fig. 1b) and a negative correlation with AR score (Fig. 1c). In the Taylor et al. 2010 dataset^31^, we found that *DPYSL5* expression correlated with *CGA*, *SYP,* and *ASCL1* expression, and reversibly correlated with AR target *PSA* (*KLK3*) expression (Supplementary Fig. 1a) suggesting that a subset of patient tumors also in the Taylor et al. dataset display neuroendocrine features. Moreover, patient tumors with high *DPYSL5* expression in the Taylor et al. 2010 dataset exhibited significantly lower disease-free survival compared to patients with low *DPYSL5* expression (Fig. 1d).

Next, to assess the expression of DPYSL5 protein in treatment-resistant patient tumors, we constructed a CRPC-t-NEPC-like tissue microarray (TMA) comprising 135 prostate cancer patient tissue samples with untreated (*n* = 37), CRPC/transurethral resection of the prostate (TURP) (*n* = 43), and t-NEPC-like (*n* = 55) samples. DPYSL5, NCAM, CGA, SYP and AR protein expressions were evaluated in the tumor tissue samples using immunohistochemistry (IHC, Fig. 1e). The results revealed that DPYSL5 was expressed in 40% of t-NEPC-like patient tumors with strong or moderate intensity (immunoscores 3/3 and 2/3). In contrast, DPYSL5 was not detectable (immunoscore 0/3) or only weakly stained (immunoscore 1/3) in most untreated patient tumors (Fig. 1f). Importantly, DPYSL5 protein expression significantly correlated with SYP (Pearson 0.4927), CGA (Pearson 0.5364) and NCAM (Pearson 0.6121) and inversely correlated with AR (Pearson −0.428) and PSA (Pearson −0.3412) (Table 1). We then performed DPYSL5 IHC staining also for patient PDX tumor cohort (Fig. 1g). A similar significant trend, as observed in patient tumor tissue samples, was detected between NEPC and adenocarcinoma or CRPC tumors in the PDX tumor TMA (Fig. 1h). These results collectively demonstrate that DPYSL5 is significantly and nearly exclusively overexpressed in treatment-induced NEPC tumors compared to untreated prostate adenocarcinoma tumors. Interestingly, our mRNA expression analysis of DPYSL5 across a panel of non-malignant somatic tissues revealed that DPYSL5 is only expressed in the central nervous system (Supplementary Fig. 1b). These results suggest that DPYSL5 may serve as a promising indicator of neuroendocrine differentiation and a potential biomarker for neuroendocrine prostate cancer.

**Table 1:** DPYSL5 IHC staining Pearson correlation with SYP, CgA, NCAM, AR and PSA in Vancouver Prostate Centre NEPC patient cohort (*n* = 55).

Correlation: DPYSL5 vs.	SYP	CgA	CD56	AR	PSA
Pearson r					
r	0.4927	0.5364	0.6121	−0.428	−0.3412
95% confidence interval	0.2617 to 0.6704	0.3160 to 0.7018	0.4119 to 0.7560	−0.6226 to −0.1836	−0.5561 to −0.08344
R squared	0.2428	0.2877	0.3747	0.1832	0.1164
P value					
P (two-tailed)	0.0001	< 0.0001	< 0.0001	0.0011	0.0108
P value summary	***	***	***	**	*
Significant? (alpha = 0.05)	Yes	Yes	Yes	Yes	Yes
Number of XY Pairs	55	55	54	55	55

### DPYSL5 expression is suppressed by androgen receptor

**Fig. 2:**
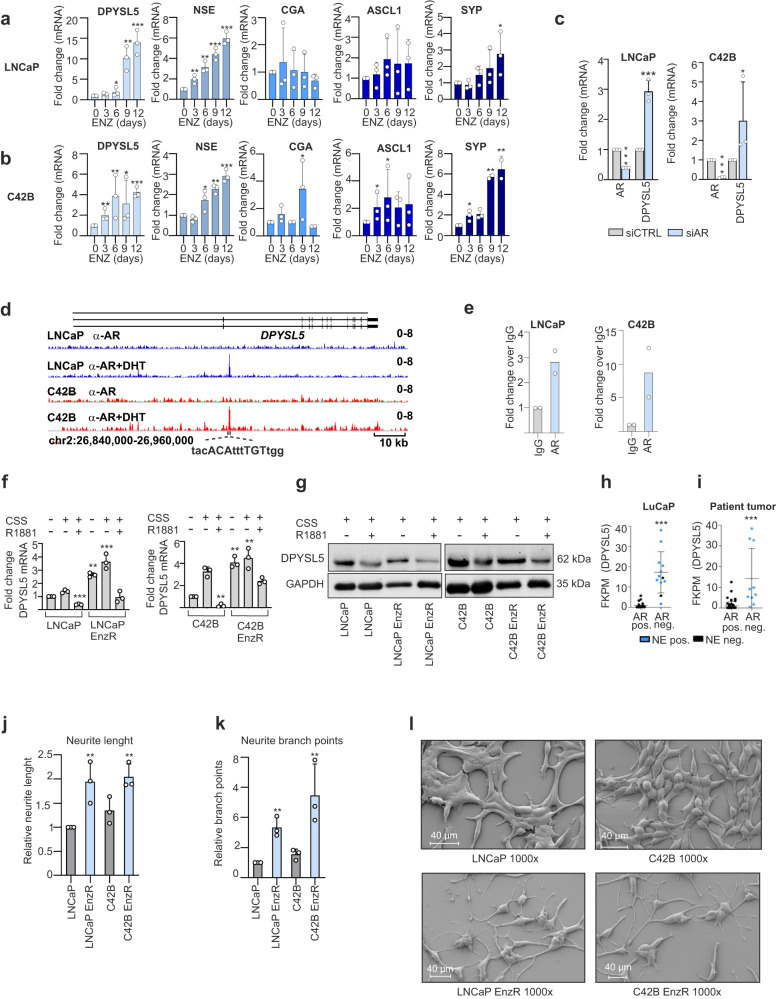
DPYSL5 expression is suppressed by AR. **a** Enzalutamide (ENZ) induces *DPYSL5* mRNA expression along with NEPC markers, *NSE* in LNCaP, and (**b**) *NSE*, *SYP,* and *ASCL1* in C42B cells. **c** Silencing *AR* expression with siRNA (siAR) in LNCaP and C42B cells increases *DPYSL5* expression based on qPCR analysis. Bars represent mean ± SD with *n* = 3. p-values shown as asterisks (*p ≤ 0.05, **p ≤ 0.01 and ***p ≤ 0.001). **d** AR ChIP-seq data from LNCaP and C42B cells stimulated with DHT. A putative AR binding site in the second intron of *DPYSL5* gene with a sequence of 5’-TACACATTTTGTTGG-3’ is highlighted. **e** AR binding to the *DPYSL5* gene was verified using AR-DPYSL5 qPCR-ChIP. **f** The effect of R1881 on *DPYSL5* mRNA levels in LNCaP and in LNCaP EnzR cells (expression compared to LNCaP cells grown with FBS) and in C42B and in C42B EnzR cells (expression compared to C42B cells grown with FBS) and (**g**) on protein level (R1881 = synthetic androgen methyltrienolone, CSS=charcoal stripped serum). **h** *DPYSL5* is highly expressed in AR-negative, NE-positive LuCaP xenografts and (**i**) patient tumor samples in Labreque et al. 2019 dataset. **j** Neurite lengths and (**k**) branch points in ENZ-resistant (EnzR) and parental LNCaP and C42B cells calculated using IncuCyte NeuroTrack analysis software module. **l** Comparison of the cellular ultrastructures of LNCaP, LNCaP EnzR, C42B and C42B EnzR cells using scanning electron microscopy.

As AR inhibitor ENZ is known to induce t-NEPC, we first exposed LNCaP and C42B prostate cancer cells to ENZ and monitored the expression of *DPYSL5* and neuronal lineage markers every three days over a total of twelve days. DMSO-exposed cells were used as a control. ENZ exposure resulted in a gradual increase in *DPYSL5* expression, reaching 12-fold and 4.5-fold mRNA levels after 12 days in LNCaP and C42B cells, respectively. This was accompanied by an increase in *NSE* mRNA in LNCaP cells and *NSE*, *SYP* and *ASCL1* mRNA in C42B cells (Fig. 2a, b). In addition, silencing AR using small interfering RNA (siAR) led to a 3-fold increase in *DPYSL5* mRNA expression after three days of silencing (Fig. 2c).

To determine whether DPYSL5 is directly regulated by AR, we analysed previously published AR ChIP-seq data from LNCaP and C42B cells stimulated with Dihydrotestosterone (DHT) to identify potential AR binding sites surrounding the DPYSL5 locus^32^. The results identified a putative AR binding site in the second intron of *DPYSL5* gene (Fig. 2d). Subsequently, we confirmed the binding of AR to this site using AR-DPYSL5 qPCR-ChIP (Fig. 2e, positive control in Supplementary Fig. 2a and negative control in Supplementary Fig. 2b). Next, we evaluated whether the activation of AR signaling affects DPYSL5 expression. First, we induced DPYSL5 expression by culturing LNCaP and C42B cells under charcoal-stripped serum (CSS) for three days and then activated the AR signaling using synthetic androgen metribolone (R1881). The results showed that CSS-induced *DPYSL5* expression was significantly suppressed by AR activation with R1881 at both mRNA (Fig. 2f) and protein levels (Fig. 2g). The expression of the AR target *PSA* (*KLK3*) was utilized to confirm the R1881-induced AR signaling (Supplementary Fig. 2c and d). Exploring data from Labrecque et al. 2019^33^, we identified high *DPYSL5 *expression in AR-negative LuCaP PDX tumors which are also positive for neuroendocrine features (Fig. 2h). In contrast, low *DPYSL5* mRNA expression was observed in AR-positive LuCaP PDX tumors (Fig. 2h). Surprisingly, we also noted in the Labrecque et al. 2019 dataset an increased DPYSL5 expression in two double- negative (AR and NE negative) prostate cancer transition stage tumors that can potentially give rise to  NEPC tumors over time^33^. Significantly elevated *DPYSL5* expression was also detected in AR-negative and NE-positive patient tumors (Fig. 2i). Taken together, these results suggest AR-mediated regulation of DPYSL5 expression.

### Enzalutamide promotes the growth of neurite-like structures

Given the involvement of DPYSL5 in axon guidance and neurite outgrowth, and our observation that ENZ induces DPYSL5 expression, we analyzed  the morphological changes in ENZ-exposed prostate cancer cells using IncuCyte S3 live-cell imaging. LNCaP and C42B cells were cultured under ENZ pressure for up to ten days, and the IncuCyte NeuroTrack software module was utilized to analyze in more detail the formed neurite-like structures, including the length and branch points of these cytoplasmic protrusions. The results showed that ENZ pressure significantly induced the growth of neurite-like structures in prostate cancer cells (Fig. 2j, k). This finding was further supported by similar observations in ENZ-exposed VCaP cells (Supplementary Fig. 2e, f). To delve deeper into these morphological changes, we analyzed cellular ultrastructure using a scanning electron microscope (SEM). The results revealed a substantial alteration in cellular ultrastructure compared to control cells; the ENZ-exposed cells exhibited a net-like structure with cytoplasmic protrusions and elongated node-like structures (Fig. 2l), aligning with our live-cell imaging results.

### DPYSL5 promotes stemness, invasiveness and t-NEPC plasticity

**Fig. 3:**
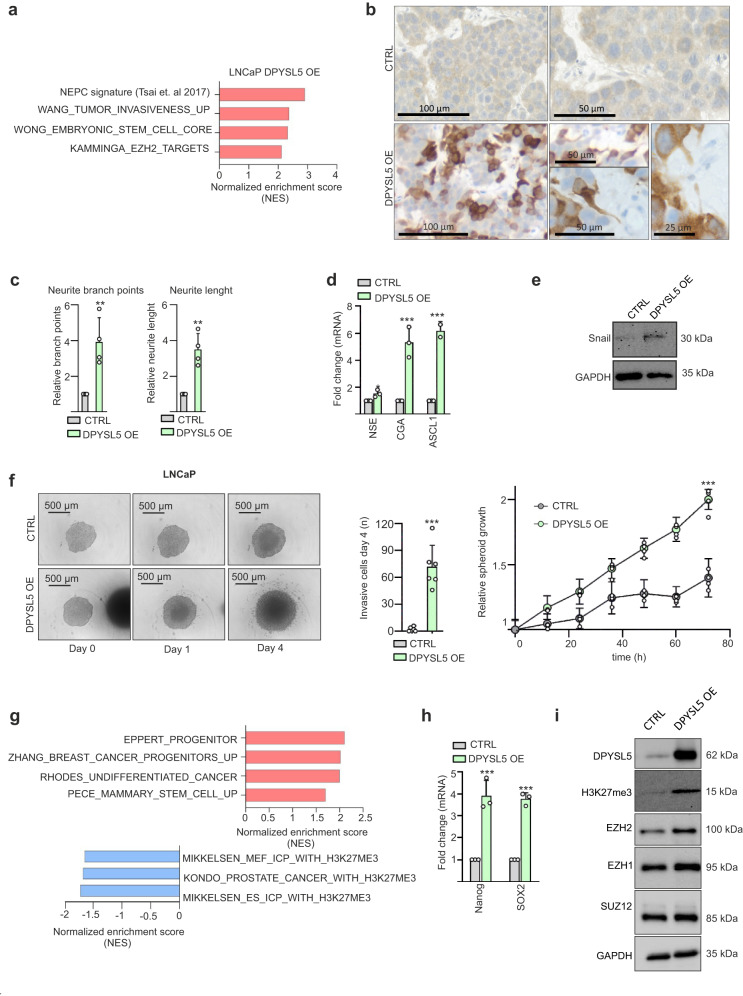
DPYSL5 promotes stemness through the activation of the PRC2 complex. **a** Gene set enrichment analysis (GSEA) reveals that DPYSL5 overexpression (DPYSL5 OE) in LNCaP cells promotes the expression of genes commonly upregulated in NEPC (NEPC-signature) and in embryonic stem cells. Additionally, upregulation of EZH2 targets and genes related to invasiveness is observed in response to DPYSL5 overexpression. (p-value > 0.001 and normalized enrichment scores (NES) > 2 were used as cut offs). **b** DPYSL5 overexpression promotes neuron-like morphology in CAM tumors in C42B cells when compared to control (CTRL) cells. Brown: DPYSL5 immunohistochemical staining. **c** DPYSL5 overexpression leads to a significant increase in the number of neurite branch points and neurite length in LNCaP cells. p-values are shown as asterisks (*p ≤ 0.05, **p ≤ 0.01 and ***p ≤ 0.001). **d** DPYSL5 overexpression increases mRNA expression of NE-markers *NSE*, *CGA* and *ASCL1* in LNCaP cells based on qPCR analysis. **e** Additionally, protein expression of Snail increases in LNCaP cells in response to DPYSL5 overexpression. **f** Spheroid formation of LNCaP CTRL cells and DPYSL5 overexpression cells. DPYSL5 overexpression promotes invasiveness and spheroid growth based on analysis using IncuCyte live-cell imaging system and the associated software. **g** DPYSL5 overexpression results in the upregulation of genes related to progenitor cells and stemness, while H3K27 trimethylation-associated genes are downregulated. **h** DPYSL5 overexpression leads to the upregulation of central stem cell markers *Nanog* and *SOX2* in LNCaP cells. **i** DPYSL5 overexpression increases protein levels of epigenetic regulators EZH2, EZH1 and SUZ12, and also of trimethylated H3K27 (H3K27me3).

To understand the molecular changes linking DPYSL5 to t-NEPC plasticity, we performed RNA-sequencing on *DPYSL5* overexpressing LNCaP cells and utilized gene set enrichment analysis (GSEA) to obtain an overview of the pathways enriched by *DPYSL5* overexpression (Fig. 3a, validation of *DPYSL5* overexpression in Supplementary Fig. 3a). The results revealed an enrichment of gene sets upregulated in NEPC patient tumors (gene set generated using patient data from Tsai et al. 2017), and upregulation of stemness (Wong_embyonic_stem_cell_core) and invasiveness (Wang_tumor_invasiveness_up) related gene sets, as well as EZH2 targets (Kamminga_EZH2_targets) in DPYSL5-overexpressed cells compared to control cells (Fig. 3a).

Since DPYSL5 is known to promote the growth of axons in early nervous system development, and based on our GSEA results showing DPYSL5 overexpression-induced NEPC signature and invasiveness, we investigated whether DPYSL5 overexpression alone can induce neurite-like changes and promote the growth of invadopodia in prostate cancer cells and in chick chorioallantoic membrane (CAM) tumors. For the CAM assay, we utilized C42B cells as they were able to form well-growing tumors. We overexpressed DPYSL5 (validation of the DPYSL5 overexpression in Supplementary Fig. 3b), and the control and DPYSL5 overexpressing cells (heterogeneous cell population including approx. 50% of DPYSL5 overexpressing cells) were implanted on the CAM.  Sections of the formed tumors were fixed and stained with H&E and DPYSL5 antibody (Supplementary Fig. 3c). The results revealed that cells overexpressing DPYSL5 showed a neurite-like morphology with long neurite-like protrusions in the tumor (Fig. 3b).

Moreover, to quantify the morphological changes in vitro, we monitored neurite length and branch points in DPYSL5 overexpressing cells using IncuCyte S3 live-cell imaging and NeuroTrack software module. The results showed that DPYSL5 overexpression leads to a significant induction of neurite length and branch points in LNCaP (Fig. 3c) as well in C42B and VCaP prostate cancer cells (Supplementary Fig. 3d, e) when compared to control (CTRL) cells.

Since our GSEA data indicated that DPYSL5 overexpression-enriched gene sets included NEPC signature and Wang_tumor_invasiveness_up gene sets, we aimed to verify, using qPCR and western blot analysis, whether DPYSL5 overexpressing LNCaP and C42B cells display NE characteristics or invasiveness. To this end, we observed increased mRNA expression of neuronal lineage markers *NSE*, *CGA,* and *ASCL1* (LNCaP: Fig. 3d, C42B: Supplementary Fig. 3f), as well as an elevated protein level of the invasion-inducing Snail (LNCaP; Fig. 3e) in DPYSL5 overexpressing cells compared to CTRL cells. Moreover, we conducted a spheroid invasion assay using Matrigel and IncuCyte S3 live-cell imaging to determine if DPYSL5 overexpression is capable of inducing invasion in 3D tumor spheroids. The growth and invasion of the spheroids were monitored every 6 h for 4 days. The results revealed that while nearly no invasive cells were detected in CTRL cells, dozens of invasive cells were seen in DPYSL5 overexpressing cells after 4 days on Matrigel (Fig. 3f). In addition, spheroids consisting of DPYSL5 overexpressing cells grew significantly faster than control spheroids (Fig. 3f).

### DPYSL5 induces neuroendocrine lineage plasticity by promoting PRC2 complex activity

In a more detailed analysis of the gene sets affected by DPYSL5 overexpression using our RNA-seq data, several stemness and progenitor-related gene sets were found (Fig. 3g). These included the gene set “MIKKELSEN_ES_ICP_WITH_H3K27ME3” which was downregulated in response to DPYSL5 overexpression (Fig. 3g), suggesting that DPYSL5 overexpression promotes a similar methylation status as seen in embryonic stem cells. The role of DPYSL5 as a stemness promoter was supported by our finding that increased mRNA expression of reprogramming factors *Nanog* and *SOX2* were detected in DPYSL5 overexpressing LNCaP (Fig. 3h) and C42B cells compared to CTRL cells (Supplementary Fig. 3g).

Based on the GSEA results indicating induced stemness, EZH2 target gene expression, and downregulation of H3K27 trimethylation-related genes, we hypothesized that DPYSL5 could promote the activity of polycomb repressive complex 2 (PRC2). To determine if DPYSL5 overexpression affects PRC2 activity and the trimethylation on H3K27 (H3K27me3), we studied the H3K27me3 status and the protein expression of the lineage reprogramming factors EZH2, EZH1 and SUZ12. Surprisingly, DPYSL5 overexpression induced both EZH2 and H3K27me3 levels indicating induced PRC2 activity (Fig. 3i). Our data thus indicates that DPYSL5 can activate PRC2 complex, as shown by increase in EZH2 and H3K27me3 levels, enrichment of EZH2 target genes, and downregulation of H3K27 trimethylation-related genes. DPYSL5 overexpression also led to increased mRNA expression of *Nanog*, *SOX2*, *NSE*, *CGA,* and *ASCL1,* suggesting that cells achieve characteristic of neuron-like cancer progenitor cells, possibly through the activation of EZH2.

### Depletion of DPYSL5 initiates a switch from lineage-committed Enzalutamide-resistant prostate cancer cells to a luminal phenotype

**Fig. 4:**
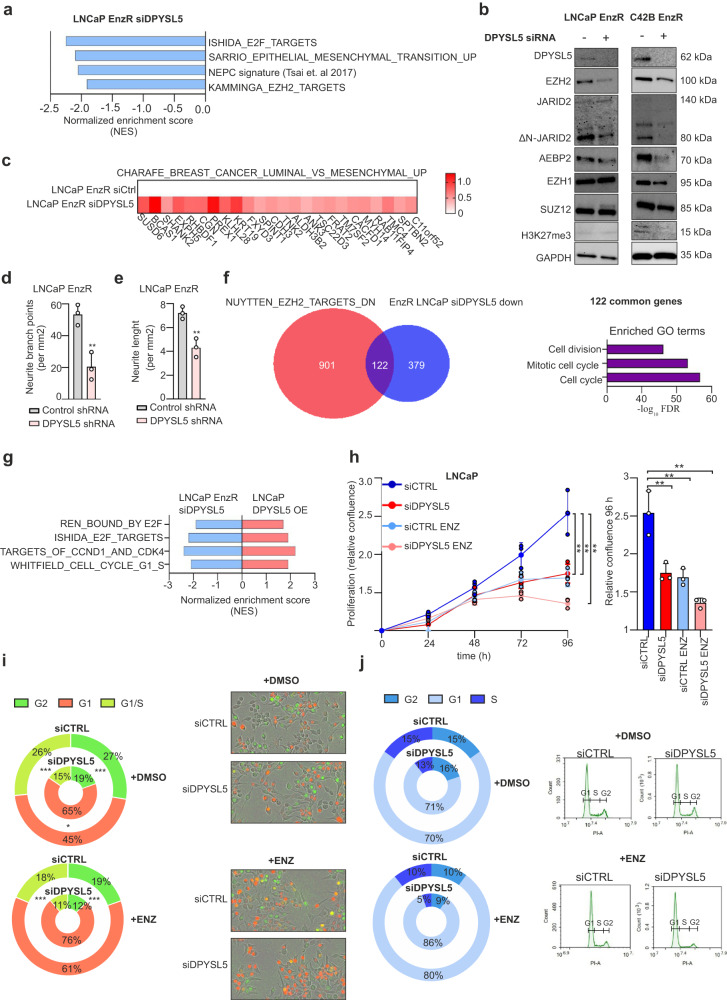
DPYSL5 is a key factor in the regulation of NEPC phenotype and cell proliferation and promotes G1 arrest. **a** GSEA reveals that DPYSL5 silencing decreases the expression of genes commonly upregulated in NEPC and epithelial mesenchymal transition in LNCaP EnzR cells. In addition, E2F and EZH2 targets are downregulated. **b** DPYSL5 silencing leads to the downregulation of EZH2 and truncated JARID2 in LNCaP EnzR cells, and to the downregulation of EZH2, truncated JARID2, EZH1, SUZ12, AEBP2, and H3K27me3 in C42B EnzR cells based on western blot analysis. **c** Upregulation of luminal markers in DPYSL5 silenced LNCaP EnzR cells. **d** DPYSL5 silencing decreases neurite branch points and (**e**) neurite length in LNCaP-EnzR cells. **f** 122 common genes involved in cell cycle and cell division were identified when genes downregulated by DPYSL5 siRNA in LNCaP EnzR cells (EnzR LNCaP siDPYSL5 down) were compared with genes downregulated by EZH2 (NYUTTEN_EZH2_TARGETS_DN). Left: Venn-diagram of the comparison. Right: Bar graph showing the gene ontology (GO) of the 122 common genes. **g** DPYSL5 regulates the expression of E2F and CCND1 targets, and cell cycle related genes (p-value > 0.001 was used as a cut off). **h** Proliferation of LNCaP siRNA control cells (siCTRL, in blue), ENZ treated LNCaP siRNA control cells (siCTRL ENZ, in light blue), DPYSL5 silenced LNCaP cells (siDPYSL5, in red) and ENZ treated, DPYSL5 silenced LNCaP cells (siDPYSL5 ENZ, in light pink) was analyzed using IncuCyte. A significant decrease in proliferation with DPYSL5 siRNA was observed, and the effect was further enhanced when combined with ENZ. Left: Proliferation curves based on confluency analysis with IncuCyte software. Right: Bar graph of the relative confluencies at 96 h timepoint. Bars represent mean ± SD with *n* = 3. p-values shown as asterisks (*p ≤ 0.05, **p ≤ 0.01 and ***p ≤ 0.001). **i** IncuCyte cell cycle analysis and (**j**) PI staining-based FACS analysis of LNCaP control siRNA cells (siCTRL +DMSO), ENZ exposed LNCaP siRNA control cells (siCTRL +ENZ), DPYSL5 silenced LNCaP cells (siDPYSL5 +DMSO) and ENZ exposed, DPYSL5 silenced LNCaP cells (siDPYSL5 +ENZ) showing an increase of cells in G1 phase in response to DPYSL5 silencing. Red: mKate-labeled Cdt1 (G1/S transition and G1 phase), Green: GFP-labeled Geminin (S/G2 transition and at G2 phase). The effect was more drastic when DPYSL5 silencing was combined with ENZ supplementation when compared to the control cells.

As increased DPYSL5 expression was capable of promoting NE lineage plasticity, we wanted to know whether DPYSL5 expression is required to maintain the NE differentiated status in ENZ-resistant cells. Thus, we performed RNA-sequencing and GSEA on ENZ-resistant (EnzR) LNCaP cells silenced with DPYSL5 siRNA and compared the results with cells silenced with non-targeting siRNA. The GSEA results showed that silencing DPYSL5 significantly downregulated genes involved in the NEPC signature, epithelial-mesenchymal transition, and E2F and EZH2 targets (Fig. 4a). Moreover, a western blot analysis of PRC2 complex members revealed that DPYSL5 depletion led to reduced protein levels of EZH2, truncated JARID2, and AEBP2 in EnzR LNCaP cells; EZH2, truncated JARID2, AEBP2, EZH1, and H3K27me3 in EnzR C42B cells (Fig. 4b); and EZH2 and truncated JARID2 in NCI-H660 cells expressing 7-fold DPYSL5 mRNA compared to LNCaP cells (Supplementary Fig. 4c, Supplementary Fig. 4d). In addition, increased levels of luminal marker expression were detected in RNA-seq data of DPYSL5 depleted LNCaP and C42B EnzR cells, suggesting a loss of differentiated neuronal-like status and reversion of the cells to their luminal identity (Fig. 4c, Supplementary Fig. 4e). Furthermore, depletion of DPYSL5 led to a significant decrease in neurite length and neurite branch points in EnzR LNCaP and EnzR C42B cells compared to controls based on IncuCyte live-cell imaging analysis (Fig. 4d, e, Supplementary Fig. 4f, g). In summary, based on the RNA-seq analysis and immunoblotting results on DPYSL5 silenced EnzR cells, DPYSL5 is an important factor in maintaining the differentiated neuronal-like status of EnzR cells, and depletion of DPYSL5 leads to the acquisition of luminal properties.

### DPYSL5 depletion leads to decreased proliferation and cell cycle arrest in G1

Our results revealed that DPYSL5 depletion downregulates EZH2 protein expression in EnzR LNCaP cells. Therefore, we took a closer look to see if genes downregulated by DPYSL5 silencing overlap with EZH2 downregulated targets (NUYTTEN_EZH2_TARGETS_DN). We discovered that the genes silenced by DPYSL5 and  the targets downregulated by EZH2 share 122 genes involved in cell cycle regulation (Fig. 4f). Regarding cell cycle-related gene sets, we found out that DPYSL5 depletion reduced the enrichment of E2F targets, CCND1, and CDK4 targets and G1/S-related cell cycle genes, while DPYSL5 overexpression promoted the enrichment of these genes (Fig. 4g). Moreover, to determine if DPYSL5 depletion potentiates the ENZ-reduced cell proliferation, we silenced DPYSL5 in LNCaP cells and monitored the cell confluence with and without ENZ using IncuCyte. The results revealed that DPYSL5 silenced cells proliferated slower than control cells, and the supplementation of ENZ on DPYSL5 silenced cells led to an additive decrease in cell proliferation (Fig. 4h). Additionally, decrease in the proliferation in the NEPC cell line NCI-H660 in response to DPYSL5 depletion was observed (Supplementary Fig. 4h).

To understand the combined effect of ENZ and DPYSL5 silencing on cell cycle phases, we generated a cell line expressing GFP-labeled Geminin and mKate-labeled Cdt1. Cdt1 expression is detected at G1 phase and in G1/S transition, while Geminin is expressed in S/G2 transition and at G2 phase, allowing analysis of the cell cycle phases with IncuCyte live- cell imaging based on the observed fluorescence signal. The results showed that fewer cells were able to achieve G1/S-phase when DPYSL5 was silenced (26% of control and 15% of DPYSL5 silenced cells in G1/S-phase), indicating that DPYSL5 depletion induces cell cycle arrest in G1 phase (Fig. 4i). Combined DPYSL5 depletion with ENZ supplementation resulted in an enhanced cell cycle arrest in G1, as 18% of control and 11% of DPYSL5 silenced cells were able to process into G1/S-phase (Fig. 4i). Furthermore, cell cycle analysis with flow cytometry using PI-stained cells showed similar results as IncuCyte live-cell imaging analysis, supporting that DPYSL5 depletion potentiates the ENZ-induced cell cycle arrest in G1 phase (Fig. 4j). Based on these results, DPYSL5 expression is required for prostate cancer cell proliferation and for the cells to progress further from G1-phase, especially under ADT.

## Discussion

The introduction of AR-pathway inhibitors for the treatment of CRPC has unfortunately increased the frequency of a highly aggressive, AR-indifferent prostate cancer known as t-NEPC  (Beltran et al.^34^). Life expectancy after NEPC diagnosis is about 7 months with no targeted treatment options available. Therefore, a better understanding of the molecular mechanisms driving t-NEPC development is essential for the design and stratification of therapeutic strategies for t-NEPC.

In this study, we report for the first time that DPYSL5 is highly and nearly exclusively expressed in treatment-induced neuroendocrine prostate cancer patient and in patient-derived xenograft tumors modeling t-NEPC. Furthermore, the expression of DPYSL5 significantly correlates with NCAM, SYP and CGA, known indicators of NEPC, and negatively correlates with AR and PSA. To our knowledge, this is the first report describing high DPYSL5 expression in t-NEPC and defining its role in driving plasticity in prostate cancer progression to treatment resistance. In addition, our results indicate that AR suppresses DPYSL5 expression by binding to the *DPYSL*5 gene locus thus explaining how ENZ-inhibited AR pathway leads to induced DPYSL5 expression, ENZ resistance, and t-NEPC phenotypic plasticity.

Though implicated in signaling during axon guidance and neurite outgrowth in neural development and regeneration, and DPYSL5 expression in other neuroendocrine cancers has been reported, the cellular functions and role of the collapsin response mediator protein DPYSL5 in prostate cancer remain largely unknown. Interestingly, exploration of RNA-seq data across a panel of non-malignant somatic tissues revealed that *DPYSL5* is primarily specific to the brain and not notably expressed in other non-malignant tissues in the body. This finding supports its great potential as a therapeutic target for t-NEPC.

Our data demonstrates that EnzR cells display a neuron-like morphology, which correlates with DPYSL5 expression. Previous studies have linked this type of morphology with neuroendocrine differentiation in prostate cancer cell lines^35–37^. We utilized live-cell imaging analysis, super-resolution microscopy, and scanning electron microscopy, and  discovered that EnzR cells form long neurite-like structures with multiple branch points. Interestingly, a similar neuron-like plasticity was observed in cells stably overexpressing DPYSL5, where these cells developed neurite-like elongated protrusions. Moreover, DPYSL5 silenced EnzR cells showed reduced length of neurite-like protrusions, branching points, and proliferation. In addition to what has been previously shown for neurons^24,25^, these results collectively indicate that DPYSL5 can induce neurite-like structures not only in neuronal tissue but also in prostate cancer cells. The clinical significance of these neurite-like structures could be linked to invasiveness, as these long, thin protrusions, also called ‘invadopodia’, have the  capability to degrade the extracellular matrix, thereby promoting metastasis^38,39^.

In addition to inducing neurite-like protrusions, DPYSL5 overexpressing cells acquired characteristics resembling those of neuron-like progenitor cancer cells, marked  by an increased expression of neuronal lineage markers *ASCL1*, *NSE,* and *CGA*, along with traits associated with stemness and invasiveness. RNA-sequencing revealed that DPYSL5 promotes H3K27 trimethylation typical to stem cells, which is facilitated through PRC2-complex. PRC2 complex activation was confirmed with western blot analyses, where increased methylation was detected alongside increased protein levels of EZH2, EZH1 and SUZ12. According to current knowledge, PRC2 activity is altered during androgen deprivation, switching the function of EZH2 from histone-methyltransferase to non-histone methyltransferase resulting in the loss of H3K27me3^40^. Consistent with this, our data showed that EnzR LNCaP cells lacked H3K27me3, suggesting that these cells are terminally differentiated. Furthermore, our EnzR LNCaP cells possessed a cleaved low molecular weight form of JARID2, which is associated with enhanced differentiation^41^. Despite these observations, EZH2 function remains crucial for NE-development, as cells do not survive AR deprivation in the absence of EZH2^42^.

Our data indicated that DPYSL5 depletion led to a reduction in EZH2 protein levels. When comparing the common genes regulated by both EZH2 and DPYSL5 silencing, we noticed that several of them were cell cycle-related. Therefore, we looked further how DPYSL5 affects cell proliferation and discovered that its  significantly decreased proliferation, particularly when combined with ENZ. In addition, DPYSL5 depletion promoted cell cycle arrest in the G1-phase, as fewer cells progressed into the S-phase. Silencing DPYSL5 also decreased the expression of E2F targets, which play a role in cell cycle regulation. Previously, Xu et al. have reported that EZH2 has a function beyond the traditional regulation of the PRC2 complex in prostate cancer, where it was shown to co-operate with E2F and regulate expression of cell cycle related genes^43^. Therefore, depleting EZH2 through DPYSL5 silencing could induce cell cycle arrest by dysregulating EZH2-E2F-driven regulation.

In addition to the reduction of EZH2 protein levels, we detected a decrease in protein levels of truncated JARID2 in response to DPYSL5 depletion. To our knowledge, this is the first time in prostate cancer where the expression of truncated JARID2 has been shown. In a prior study, truncated JARID2 has been linked with lineage-committed keratinocytes, which have a high intracellular Ca^2+^ concentration^41^. Truncated JARID2 lacks the PRC2-interacting domain and promotes the activation of genes involved in differentiation. As DPYSL5 silencing led to a decrease in genes involved in epithelial-mesenchymal transition in EnzR cells, an increased expression of luminal markers, loss of neuron-like phenotype and decreased amount of truncated JARID2, we suggest that DPYSL5 expression is required to maintain the differentiated state of lineage-committed EnzR cells.

However, the exact mechanism how DPYSL5 regulates these proteins is not clear. In glioblastoma, DPYSL5 has been previously shown to protect Notch receptors from E3 ubiquitin ligase Itch-mediated lysosomal degradation, leading to sustained Akt activation^28^. Therefore, DPYSL5 could similarly prevent also EZH2 from lysosomal degradation, as EZH2 protein levels decreased when DPYSL5 was silenced and increased when DPYSL5 was overexpressed.

Taken together, we describe here for the first time that DPYSL5 is highly overexpressed and a potential novel biomarker and therapeutic target for highly aggressive t-NEPC. Our results suggest that DPYSL5 may play an important role in t-NEPC development and that DPYSL5 expression is required for prostate cancer cell survival under androgen deprivation, making it an interesting and potential target for drug development. We hypothesize that the AR/DPYSL5/EZH2/PRC2 axis is a novel mechanism underlying prostate cancer progression to t-NEPC, and it is important to further elucidate the theranostic potential of DPYSL5 and explore any pharmacological strategies that could manipulate its expression.

## Methods

### In silico analyses

Median RNA-seq RNA expression values and clinicopathological data from prostate cancer were analyzed using cBioPortal^44,45^. GTEx Portal (gtexportal.org) was utilized to analyse the tissue-specific gene expression of DPYSL5 across the human body.

### Tissue microarray construction

Formalin fixed paraffin embedded (FFPE) tumor tissue blocks (CRPC, NEPC, non-prostate tumors) and respective non-malignant tissue blocks were retrieved from the Department of Pathology at Vancouver General Hospital (Vancouver, Canada). Tissue microarrays (TMA) of three cohorts were constructed from the following clinical samples: a NEPC discovery cohort, a NEPC-CRPC validation cohort, and a panel of a total of 45 different kinds of cancer types and respective non-malignant tissue.

FFPE of patient-derived xenografts (PDX) of prostate tumors with neuroendocrine transdifferentiation were graciously provided by the Vancouver Prostate Centre (YW and DL, Vancouver, Canada). To identify tumor areas, H&E slides of retrieved FFPE tissue blocks were reviewed and annotated by pathologists. Tissue cores from donor FFPE blocks (representative of tumor or non-malignant areas, diameter = 1 mm) were assembled in duplicates into a recipient paraffin block with the semi-automated tissue arrayer TMArrayer (Pathology Devices).

### Immunohistochemistry

Protein expression of DPYSL5 was assessed in situ with immunohistochemistry. The automated staining platform DISCOVERY ULTRA (Ventana Medical Systems) was used. Briefly, antigen retrieval was performed with Cell Conditioning 1 (CC1) (Ventana) at 95^o^C for 64 min. Slides were incubated with DPYSL5 antibody (CR-3, MA3-700, monoclonal, 1:200, ThermoFisher Scientific) at room temperature for 1 h. Slides were then incubated with AffiniPure Rabbit AntiRat IgG (H + L) (312-005-045, polyclonal, 1:1000, Jackson ImmunoResearch Laboratories) at 37^o^C for 32 min. To visualize bound antibodies, UltraMap anti-Rb HRP and the ChromoMap DAB kit (Ventana) were used. Digital images of all immunohistochemically stained slides were acquired with SCN400 Slide Scanner (Leica Microsystems). To analyze protein expression, digital scans of stained tissue were scored with Aperio ImageScope (Leica Biosystems) by an experienced pathologist (LF).

### Cell culture

Human prostate adenocarcinoma cell lines LNCaP and LNCaP C42B (C42B) were cultured in RPMI-1640 medium (Sigma) with 2 mM L-glutamine (Lonza), 10% fetal bovine serum (Gibco) and streptomycin/penicillin. NCI-H660 cell line was cultured in HITES medium. Treatment-induced NE-like Enzalutamide-resistant LNCaP-EnzR and C42B-EnzR cells were constantly cultured with 10 µM Enzalutamide.

### Quantitative PCR and RNA-seq analysis

Total RNA was extracted from cells using TRIzol (Invitrogen). RNA was reverse transcribed to cDNA with Transcriptor First Strand cDNA synthesis Kit (Roche) according to manufacturer’s instructions. Gene expression was analyzed using LightCycler 480 SYBR Green I Master (Roche), LightCycler 480 II (Roche) and 2^-ΔΔ^Ct method. Specific primers are listed in Supplementary Table 1. For RNA-sequencing, Qiagen RNEasy MiniKit was used to extract RNA and RIN-values were determined with 2100 Bioanalyzer (Agilent) RNA-seq libraries were prepared using NEBNext Poly(A) mRNA Magnetic Isolation Module (New England BioLabs, E7490) and NEBNext Ultra II Directional RNA Library Prep with Sample Purification Beads Kit (New England BioLabs, E7765). Pooled libraries were sequenced with NextSeq 500 at The EMBL Genomics Core Facility (Heidelberg, Germany). Sequenced raw reads were quality controlled, the differential transcription was analyzed as described previously^46^.

### Gene set enrichment analysis

Expression datasets were subjected to gene set enrichment analysis with GSEA (Subramanian, Tamayo, et al. (2005, PNAS) and Mootha, Lindgren, et al. (2003, Nature Genetics). Gene-Set Enrichment Analysis (GSEA) was performed using GSEA software (v.4.1.0) from the Broad Institute (Massachusetts Institute of Technology). The tool was run in classic mode to identify significantly enriched pathways. Pathways enriched with a nominal p-value < 0.05 and false discovery rate (FDR) < 0.25 were considered to be significant.

### Western Blot

Total protein from cells was extracted using SDS reagent containing proteinase inhibitor by sonication. Proteins were separated with SDS-PAGE and transferred to nitrocellulose membranes. Primary antibodies were incubated in 4^o^C overnight and HRP-conjugated secondary antibodies were incubated for 1 h in RT. The proteins were detected with ECL reagent (BioRad). For PRC2 complex, used antibodies were provided in Polycomb Group 2 (PRC2) Antibody Sampler Kit #62083 (Cell Signaling Technology), for Snail (C15D3) Rabbit mAb #3879 (Cell Signaling Technology) was used, for GAPDH (FL-335): sc-25778 (Santa Cruz Biotechnology) was used and for DPYSL5 CR-3, MA3-700 (Thermo Fisher Scientific) was used. All antibodies were diluted 1:1000. Full membrane pictures of blots are available in Supplementary Figs. 5, 6 and 7. To confirm equal load of protein on membranes, GAPDH (and Ponceau S (Sigma) for NCI-H660) was run on each individual membrane/gel that was used in the study after stripping membrane with Restore^TM^ Western Blot Stripping Buffer (Thermo Fisher Scientific). Full membrane figures of loading controls are available in Supplementary Figs. 8 and 9.

### Chromatin immunoprecipitation

Chromatin immunoprecipitation (ChIP) was performed as previously described^47^. Briefly, LNCaP and C42B cells were seeded on 10 cm plates and chromatin was fragmented to an average size of 300–500 bp by sonication (Bioruptor, UCD-300, Diagenode). Antibodies were coupled to magnetic protein G beads (Dynabeads, Invitrogen) for 16 h, sonicated lysates were incubated with antibody-coupled beads for 16 h. Antibodies used per IP: AR K183^48^, 2 µl; normal rabbit IgG (sc-2025, Santa Cruz Biotechnology), 1 µg. After extensive washes, the IP samples were reverse cross-linked in the presence of proteinase K, and DNA was purified with Monarch DNA purification kit (T1030, New England Biolabs), according to instructions. Quantitative PCR analyses were carried out with LightCycler 480 SYBR green I Master (50-7203180, Roche). Results were calculated using the formula 2−(ΔCt) × 19, where ΔCt is Ct(ChIP-template) – Ct(Input). Results are presented as fold increases over the value determined for IgG-precipitated samples. 

### Proliferation analysis

Cells were reverse transfected with 25 nM of siDPYSL5 or non-targeting control siRNA (siCTRL, ON-TARGETplus SMARTpool siRNA, Dharmacon) using OPTI-MEM and RNAiMAX (Invitrogen) and seeded on a 96-well plate. After 24 h of incubation, Enzalutamide (10 µM) or DMSO control was added and the plate was inserted to IncuCyte S3 live-cell analysis system (Sartorius; Ann Arbor, MI). Plates were imaged every day and confluence was determined from images taken by using IncuCyte S3 analysis software.

### Cell cycle analysis using flow cytometry

For the cell cycle analysis, LNCaP cells were reverse transfected with siRNA and cultured on a 6-well plate. The next day, DMSO control or Enzalutamide was added for 3 days. Cells were fixed with 70% ethanol overnight in +4^o^C. Ethanol was removed by centrifuging and cells were resuspended in PBS and incubated with RNase (150 µg/ml) and propidium iodide (15 µg/ml) in +37 ^o^C one hour protected from light. DNA content was analyzed with NovoCyte Quanteon flow cytometer and cell cycle phases were determined with NovoExpress Software.

### Cell cycle analysis using IncuCyte

The LNCaP plated on a 48-well at seeding density of 10 000 cells/well for lentiviral transductions with Incucyte Cell Cycle Green/Red Lentivirus Reagent (Cat. no. 4779, Sartorius). The cells were transduced using MOI 2.5 and after one week of culturing puromycin at concentration 1 µg/ml was used to select only successfully transduced cells. For the live cell imaging analysis on a 96-well plate a seeding density of 5000 cells/well was used and cells were siRNA transfected using reverse transfection method. Cells were imaged and the red and green fluorescent cells were quantified using Incucyte S3 and the associated analysis software.

### Neurite outgrowth measurements and live-cell imaging

For neurite length measurements, phase-contrast and green fluorescence light microscopy images were acquired using the IncuCyte S3 live-cell analysis system equipped with a 20X objective (Sartorius; Ann Arbor, MI).

### 3D Spheroid invasion assay

LNCaP cells were plated on 96-well round bottom ultra-low attachment plate (2000 cells/well) and plate was centrifuged 125 g 10 min in room temperature. After 3 days when spheroid had formed, ice-cold Matrigel was added on pre-chilled plate with final concentration of 4,5 mg/ml. Plate was centrifuged 300 g for 3 min in 4^o^C and plate was placed in 37^o^C incubator for 30 min to polymerize Matrigel. Plate was imaged every 6 h for 4 days using IncuCyte S3, and Spheroid Analysis Software Module (Sartorius; Ann Arbor, MI) was utilized to quantify the invasive cells and spheroid size.

### Scanning electron microscopy

The cells grown on coverslips were fixed with 2% glutaraldehyde and routinely dehydrated in ascending series of ethanol and hexamethyldisilazane and coated with a thin layer of gold. After processing, cells were imaged with a Zeiss Sigma HD | VP (Carl Zeiss Microscopy GmbH, Oberkochen, Germany) scanning electron microscope operated at 3 kV.

### Lentivirus vector transductions

Plasmid overexpressing DPYSL5 was created by amplifying DNA sequence of DPYSL5 from commercial plasmid with DPYSL5 in a pDNR-Dual vector backbone (DNASU) and inserted to pLVX-IRES-ZsGreen1 (Takara) using In-Fusion HD Cloning Kit (Clontech). For silencing DPYSL5, shRNA plasmid (SMARTvector Lentiviral Human DPYSL5 shRNA) was purchased from Dharmacon. Lentiviral vectors were produced by standard calcium phosphate transfections method. DPYSL5 overexpression vector was introduced to LNCaP and C42B cells using multiplicity of infection (MOI) of 40 and cells were monitored in IncuCyte S3 for one week before sample collection. shDPYSL5 was introduced to LNCaP-EnzR and C42B-EnzR with MOI of 30 and cells were monitored for one week in IncuCyte S3.

### siRNA transfections

LNCaP-EnzR, C42B-EnzR and NCI-H660 cells were plated on 6-well plates and reverse transfected with 25 nM of siDPYSL5 or siCTRL (ON-TARGETplus SMARTpool siRNA, Dharmacon) using OPTI-MEM and RNAiMAX (Invitrogen). Medium was changed after 4 h of incubation and samples were collected after 72 h.

### Chick chorioallantoic membrane (CAM) assay

Fertilized white Leghorn chicken eggs were incubated at 37 °C. Separation of the CAM was induced on embryo development day 4 (EDD4) by piercing the eggshell. On EDD8 transfected cells were collected and implanted on the CAM (10^6^ cells per egg in 1:1 PBS - Matrigel® Matrix (Corning, USA)). On EDD13, the tumors were photographed *in ovo* and excised. Tumor histology by hematoxylin-eosin (HE) and immunohistochemical staining were done as previously described^49,50^. For immunohistochemical staining, nonspecific binding was blocked with 1.5% normal rabbit serum in PBS and a rat primary antibody (Thermo MA3-700, 1:1000) and biotinylated secondary antibody (anti‐rat IgG BA4000, Vector Laboratories, Burlingame, CA) (1:200 in 1.5% normal rabbit serum) were used. Stained sections were viewed with Zeiss Axio Imager A2 light microscope (Carl Zeiss Microimaging GmbH, Zeiss, Jena, Germany). For image capturing the stained sections were scanned by Nanozoomer XR digital slide scanner (Hamamatsu Photonics K.K., Hamamatsu City, Japan) at 40× and images captured with NDP view 2 software (Hamamatsu Photonics K.K) provided by the Biobank of Eastern Finland.

### Statistics and reproducibility

Statistical analyses were carried out using the GraphPad Prism version 5.00 for Windows, (GraphPad Software, San Diego, CA, USA). For qPCR, t-test was used. Differences were considered significant when *p ≤ 0.05, **p ≤ 0.01 and ***p ≤ 0.001. Error bars are presented as SD-values. Representative data has been repeated at least twice with a minimum of two independent biological samples and three technical replicates.

### Reporting summary

Further information on research design is available in the Nature Portfolio Reporting Summary linked to this article.

### Supplementary information

Supplementary Information

Description of Additional Supplementary Files

Supplementary Data 1

Reporting Summary

## Data Availability

Generated RNA‑sequencing datasets supporting the conclusions of this article have been submitted to the NCBI Gene Expression Omnibus database (http://www.ncbi.nlm.nih.gov/geo/); accession code: GSE151433. Other data generated in this study are included in Supplementary Data 1.

## References

[1] Aggarwal R (2018). Clinical and genomic characterization of treatment-emergent small-cell neuroendocrine prostate cancer: a multi-institutional prospective. Study J. Clin. Oncol..

[2] Wang HT (2014). Neuroendocrine Prostate Cancer (NEPC) progressing from conventional prostatic adenocarcinoma: factors associated with time to Development of NEPC and Survival From NEPC Diagnosis—A Systematic Review and Pooled Analysis. J. Clin. Oncol..

[3] Vlachostergios PJ, Papandreou CN (2015). Targeting neuroendocrine prostate cancer: Molecular and clinical perspectives. Front. Oncol..

[4] Soundararajan R, Paranjape AN, Maity S, Aparicio A, Mani SA (2018). EMT, stemness and tumor plasticity in aggressive variant neuroendocrine prostate cancers. Biochim. Biophys. Acta - Rev. Cancer.

[5] Beltran H (2016). Divergent clonal evolution of castration resistant neuroendocrine prostate cancer HHS public access author manuscript. Nat. Med..

[6] Beltran H (2011). Molecular characterization of neuroendocrine prostate cancer and identification of new drug targets. Cancer Discov..

[7] Rubin MA (2015). Concurrent AURKA and MYCN gene amplifications are harbingers of lethal treatmentrelated neuroendocrine prostate cancer. Neoplasia.

[8] Gleave ME (2016). The master neural transcription factor BRN2 is an androgen receptor–suppressed driver of neuroendocrine differentiation in prostate. Cancer Cancer Discov..

[9] Dang Q (2015). Anti-androgen enzalutamide enhances prostate cancer neuroendocrine (NE) differentiation via altering the infiltrated mast cells → androgen receptor (AR) → miRNA32 signals. Mol. Oncol..

[10] Deeble PD, Murphy DJ, Parsons SJ, Cox ME (2002). Interleukin-6- and Cyclic AMP-mediated signaling potentiates neuroendocrine differentiation of LNCaP prostate tumor cells. Mol. Cell. Biol..

[11] Ku SY (2017). Rb1 and Trp53 cooperate to suppress prostate cancer lineage plasticity, metastasis, and antiandrogen resistance. Science.

[12] Akamatsu S (2015). The placental gene PEG10 promotes progression of neuroendocrine prostate cancer. Cell Rep..

[13] Zhang X (2015). SRRM4 expression and the loss of REST activity may promote the emergence of the neuroendocrine phenotype in castration-resistant prostate cancer. Clin. Cancer Res..

[14] Ketola K (2017). Targeting prostate cancer subtype 1 by Forkhead box M1 pathway inhibition. Clin. Cancer Res..

[15] Jennifer, A. F., Joseph, E. S., Saba, T., Isla, B. & Amy, V. P. hASH1 nuclear localization persists in neuroendocrine transdifferentiated prostate cancer cells, even upon reintroduction of androgen. 1–15 10.1038/s41598-019-55665-y (2019). 10.1038/s41598-019-55665-y PMC6911083 31836808

[16] Nouruzi S (2022). ASCL1 activates neuronal stem cell-like lineage programming through remodeling of the chromatin landscape in prostate cancer. Nat. Commun..

[17] Clermont PL (2015). Polycomb-mediated silencing in neuroendocrine prostate cancer. Clin. Epigenetics.

[18] Kleb B (2016). Differentially methylated genes and androgen receptor re-expression in small cell prostate carcinomas. Epigenetics.

[19] Kim J (2018). Polycomb- and Methylation-Independent Roles of EZH2 as a Transcription Activator Polycomb- and Methylation-Independent Roles of EZH2 as a Transcription Activator. CellReports.

[20] Dardenne E (2017). N-Myc induces an EZH2-mediated transcriptional program driving Neuroendocrine. Prostate Cancer.

[21] Rao, R. A., Dhele, N., Cheemadan, S. & Ketkar, A. Ezh2 mediated H3K27me3 activity facilitates somatic transition during human pluripotent reprogramming. 1–10 10.1038/srep08229 (2015). 10.1038/srep08229 PMC4316165 25648270

[22] Brot S (2010). CRMP5 interacts with tubulin to inhibit neurite outgrowth, thereby modulating the function of CRMP2. J. Neurosci..

[23] Tsim TY, Wong EYK, Leung MS, Wong CC (2004). Expression of axon guidance molecules and their related genes during development and sexual differentiation of the olfactory bulb in rats. Neuroscience.

[24] Ji, Z. et al. Spastin Interacts with CRMP5 to Promote Neurite Outgrowth by Controlling the Microtubule Dynamics. 1191–1205 10.1002/dneu.22640 (2018). 10.1002/dneu.22640 30257070

[25] Yamashita N (2011). CRMP5 (Collapsin Response Mediator Protein 5) Regulates Dendritic Development and Synaptic Plasticity in the Cerebellar Purkinje. Cells.

[26] Meyronet D (2008). Extensive expression of collapsin response mediator protein 5 (CRMP5) is a specific marker of high-grade lung neuroendocrine carcinoma. Am. J. Surg. Pathol..

[27] Qiu F, Yu L, Liu D, Wu Y, Qiu F (2021). CRMP5 regulates cell proliferation and development of colorectal cancer via MAPK-dependent signaling. Oncol. Lett..

[28] Moutal A (2015). CRMP5 controls glioblastoma cell proliferation and survival through Notch-dependent signaling. Cancer Res.

[29] Kaarijärvi R, Kaljunen H, Ketola K (2021). Molecular and functional links between neurodevelopmental processes and treatment-induced neuroendocrine plasticity in prostate cancer progression. Cancers (Basel).

[30] Abida W (2019). Genomic correlates of clinical outcome in advanced prostate cancer. Proc. Natl Acad. Sci. USA..

[31] Taylor BS (2010). Integrative genomic profiling of human prostate cancer. Cancer Cell.

[32] Decker KF (2012). Persistent androgen receptor-mediated transcription in castration-resistant prostate cancer under androgen-deprived conditions. Nucleic Acids Res..

[33] Labrecque MP (2019). Molecular profiling stratifies diverse phenotypes of treatment-refractory metastatic castration-resistant prostate cancer. J. Clin. Invest..

[34] Beltran, H. et al. Challenges in recognizing treatment-related neuroendocrine prostate cancer. *J. Clin. Oncol.* **30**, e386–389 (2012). 10.1200/JCO.2011.41.5166 23169519

[35] Farini D, Puglianiello A, Mammi C, Siracusa G, Moretti C (2003). Dual effect of pituitary adenylate cyclase activating polypeptide on prostate tumor LNCaP cells: Short and long-term exposure affect proliferation and neuroendocrine differentiation. Endocrinology.

[36] Yuan TC (2006). Androgen deprivation induces human prostate epithelial neuroendocrine differentiation of androgen-sensitive LNCaP cells. Endocr. Relat. Cancer.

[37] Zhu Y (2014). Interleukin-6 induces neuroendocrine differentiation (NED) through suppression of RE-1 silencing transcription factor (REST). Prostate.

[38] Daly C (2020). Tks5 SH3 domains exhibit differential effects on invadopodia development. PLoS One.

[39] Manuelli V (2022). Invadopodia play a role in prostate cancer progression. BMC Cancer.

[40] Luo J (2019). LncRNA-p21 alters the antiandrogen enzalutamide-induced prostate cancer neuroendocrine differentiation via modulating the EZH2/STAT3 signaling. Nat. Commun..

[41] Al‐Raawi D (2019). A novel form of JARID2 is required for differentiation in lineage‐committed cells. EMBO J..

[42] Bai Y (2019). Inhibition of enhancer of zeste homolog 2 (EZH2) overcomes enzalutamide resistance in castration-resistant prostate cancer. J. Biol. Chem..

[43] Xu H (2016). Integrative analysis reveals the transcriptional collaboration between EZH2 and E2F1 in the regulation of cancer-related gene expression. Mol. Cancer Res..

[44] Gao J (2013). Integrative analysis of complex cancer genomics and clinical profiles using the cBioPortal. Sci. Signal..

[45] Cerami E (2012). The cBio Cancer Genomics Portal: An open platform for exploring multidimensional cancer genomics data. Cancer Discov..

[46] Lempiäinen, J. K. et al. BCOR-coupled H2A monoubiquitination represses a subset of androgen receptor target genes regulating prostate cancer proliferation. *Oncogene* 2391–2407 10.1038/s41388-020-1153-3 (2020). 10.1038/s41388-020-1153-3 31925334

[47] Paakinaho. V., Kaikkonen, S., Makkonen, H., Benes, V. & Palvimo, J. J. SUMOylation regulates the chromatin occupancy and anti-proliferative gene programs of glucocorticoid receptor. *Nucleic Acids Res.* **42**, 1575–1592 (2014). 10.1093/nar/gkt1033 PMC3919585 24194604

[48] Karvonen, U., Kallio, P. J., Jänne, O. A. & Palvimo, J. J. Interaction of androgen receptors with androgen response element in intact cells. Roles of amino- and carboxyl-terminal regions and the ligand. *J. Biol. Chem.* **272**, 15973–15979 (1997). 10.1074/jbc.272.25.15973 9188499

[49] Härkönen K (2019). CD44s assembles hyaluronan coat on filopodia and extracellular vesicles and induces tumorigenicity of MKN74 gastric carcinoma cells. Cells.

[50] Voutilaineni K (2003). Versican in epithelial ovarian cancer: Relation to hyaluronan, clinicopathologic factors and prognosis. Int. J. Cancer.

