# Supplementary material for: DPYSL5 is highly expressed in treatment-induced neuroendocrine prostate cancer and promotes lineage plasticity via EZH2/PRC2
Source: Commun Biol. 2024 Jan 18;7:108. doi: 10.1038/s42003-023-05741-x (PMC10796342; doi:10.1038/s42003-023-05741-x)
Supplement: Supplementary file 1 — Supplementary Information [file 42003_2023_5741_MOESM1_ESM.pdf]

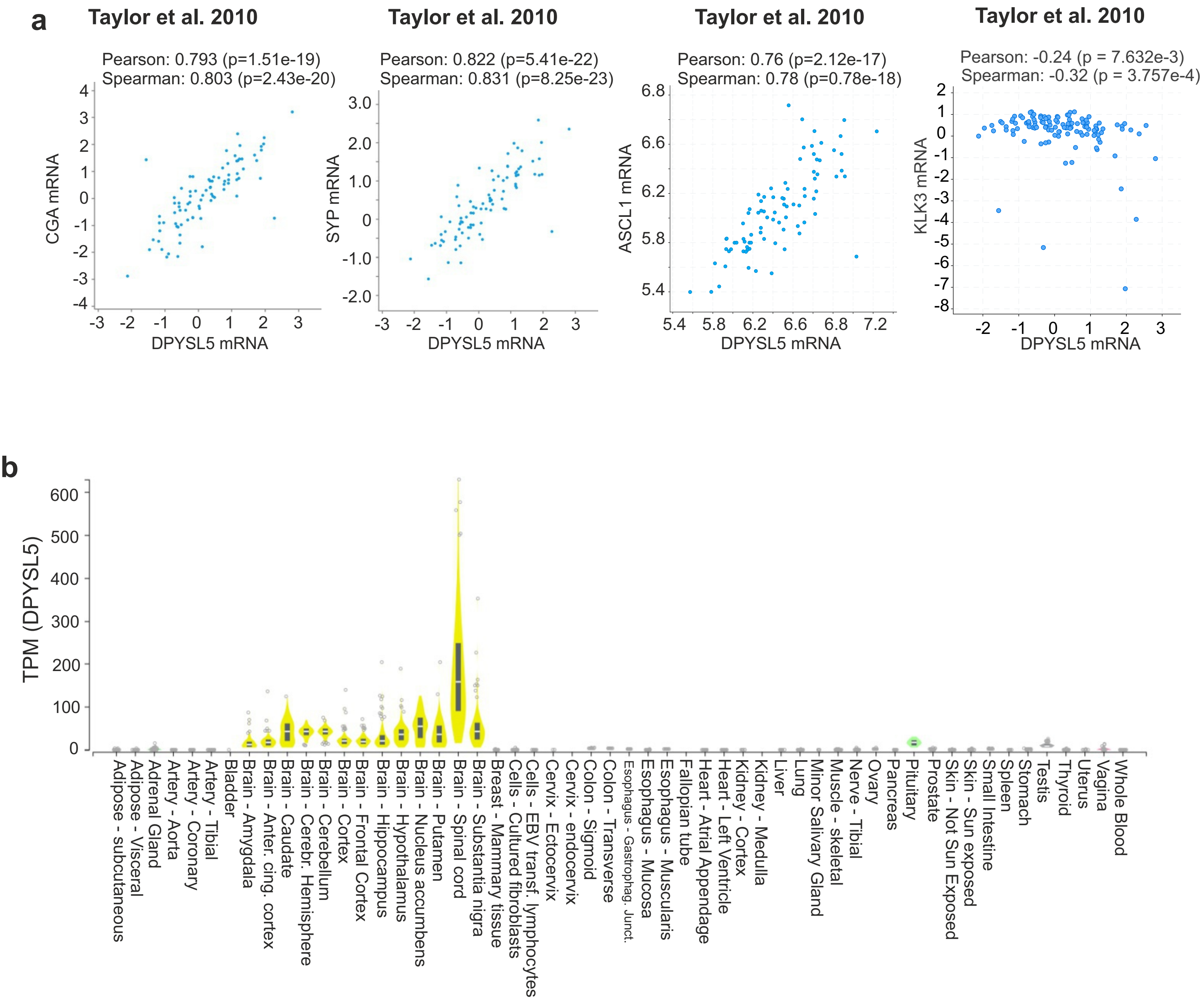

Supplementary Figure 1. a) DPYSL5 mRNA expression correlates with mRNA expression of NE markers CGA, SYP and ASCL1 while negative correlation with KLK3 mRNA expression was observed in prostate cancer patient tumors in the Taylor et al. dataset. b) DPYSL5 expression in various tissues across the body analysed using GTEx Portal (gtexportal.org). Based on this DPYSL5 is mainly expressed in the brain.

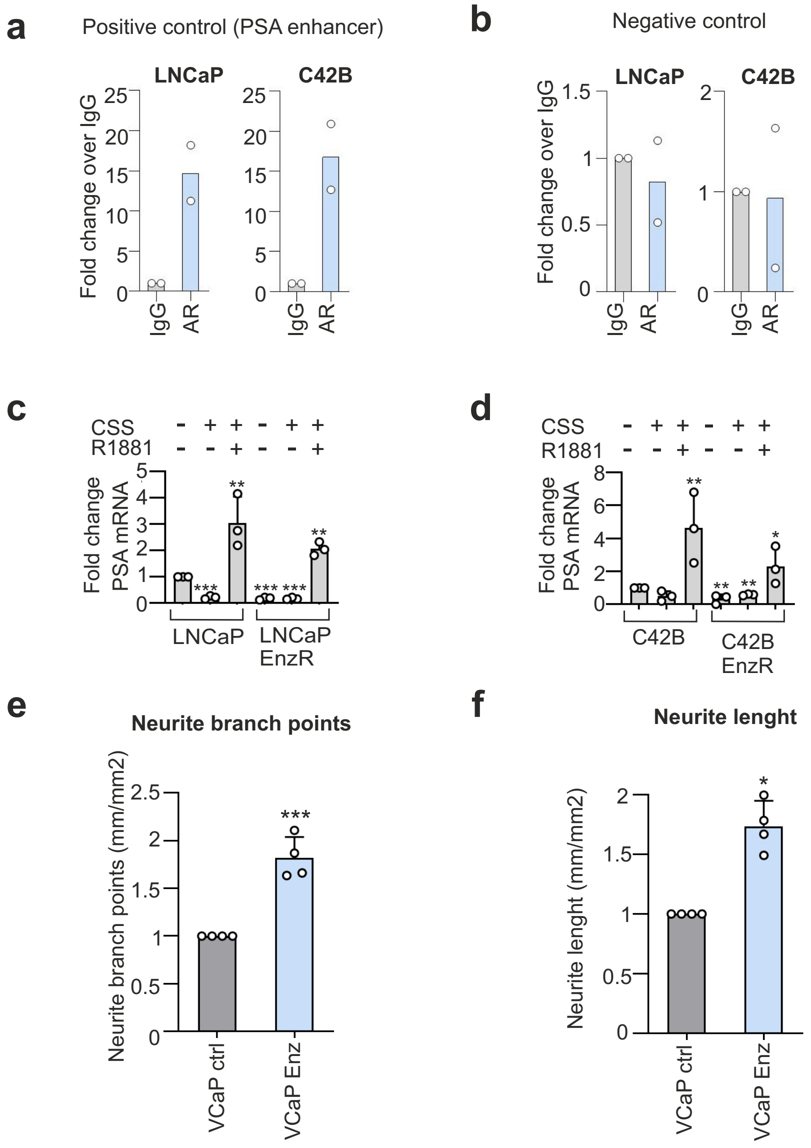

Supplementary Figure 2. a) Validation of AR binding in LNCaP and C42B cells stimulated with DHT. AR binding into PSA enhancer region was verified using AR-qPCR-ChIP with PSA enhancer specific primers. Increased binding was detected. b) Validation of AR-qPCR-ChIP assay specificity with negative genomic region primers. No significant binding was detected c) The effect of R1881 on PSA mRNA levels in d) LNCaP and in LNCaP EnzR cells (expression compared to LNCaP cells grown with FBS) and in C42B and in C42B EnzR cells (expression compared to C42B cells grown with FBS) (R1881 = synthetic androgen methyltrienolone, CSS=charcoal stripped serum). ENZ affected e) neurite branch points and f) neurite length in VCaP cells assessed using IncuCyte Neurotrack module.

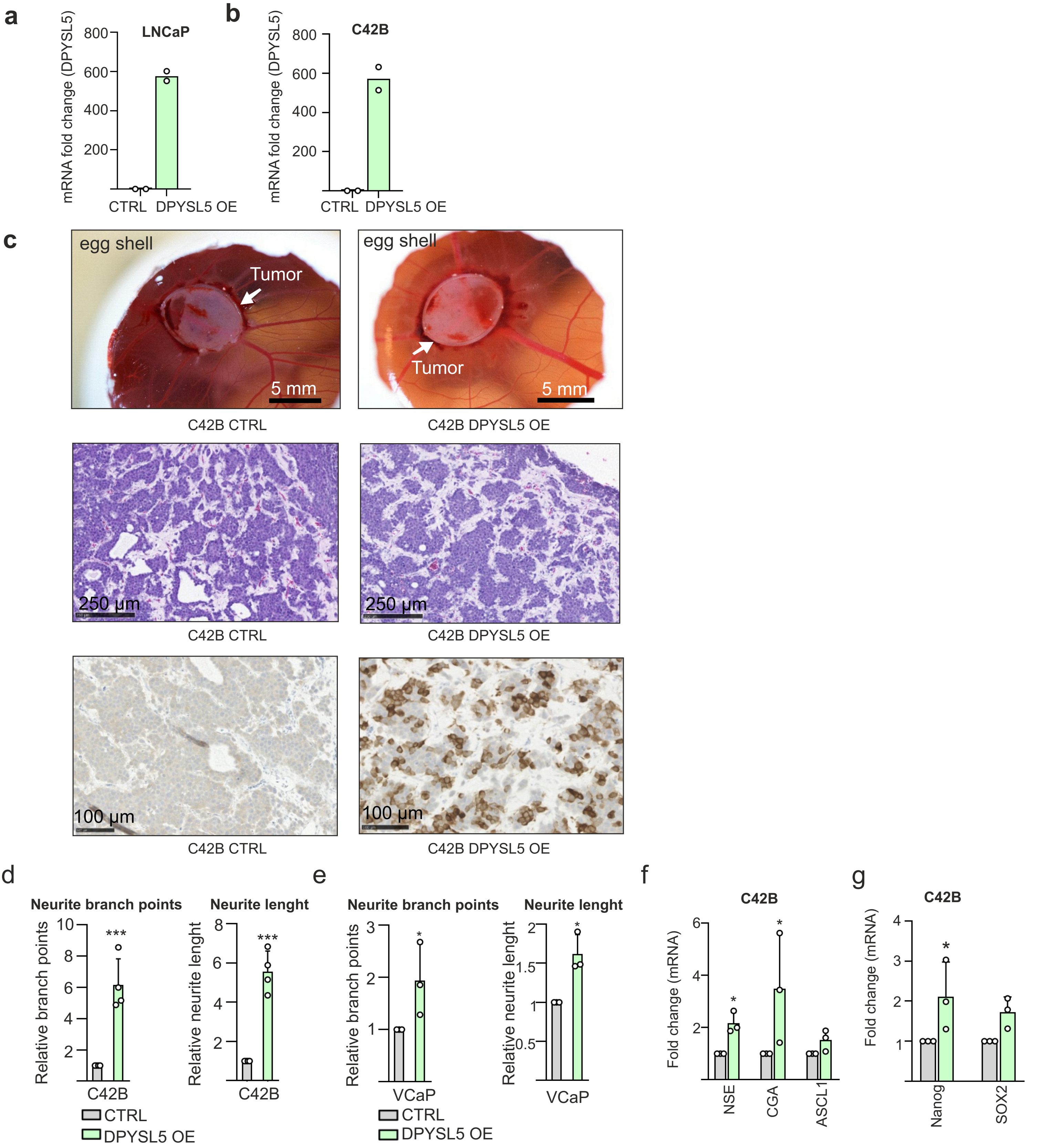

Supplementary Figure 3. a) DPYSL5 overexpression on mRNA level was verified using qPCR in LNCaP cells and in b) C42B cells. c) Top: Typical examples of C42B-derived CAM tumor (EDD13) from control cells (C42B ctrl) or from DPYSL5 overexpressing cells (C42B DPYSL5 OE). Middle: Whole sections of HE-stained CAM tumors from C42B control and C42B DPYSL5 OE cells. Bottom: Images from C42B control and C42B DPYSL5 OE CAM tumors stained with DPYSL5 antibody. d) DPYSL5 overexpression induces the formation of branch points and neurite length in C42B cells based on IncuCyte analysis. e) Similar phenomenan is observed in VCaP cells. f) DPYSL5 overexpression in C42B cells increases the mRNA expression of NE markers NSE and CGA based on qPCR analysis. g) Also the expression of stem cell markers Nanog and SOX2 are induced on mRNA level in response to DPYSL5 overexpression. p-values shown as asterisks (*p≤ 0.05, **p ≤ 0.01 and ***p ≤ 0.001).

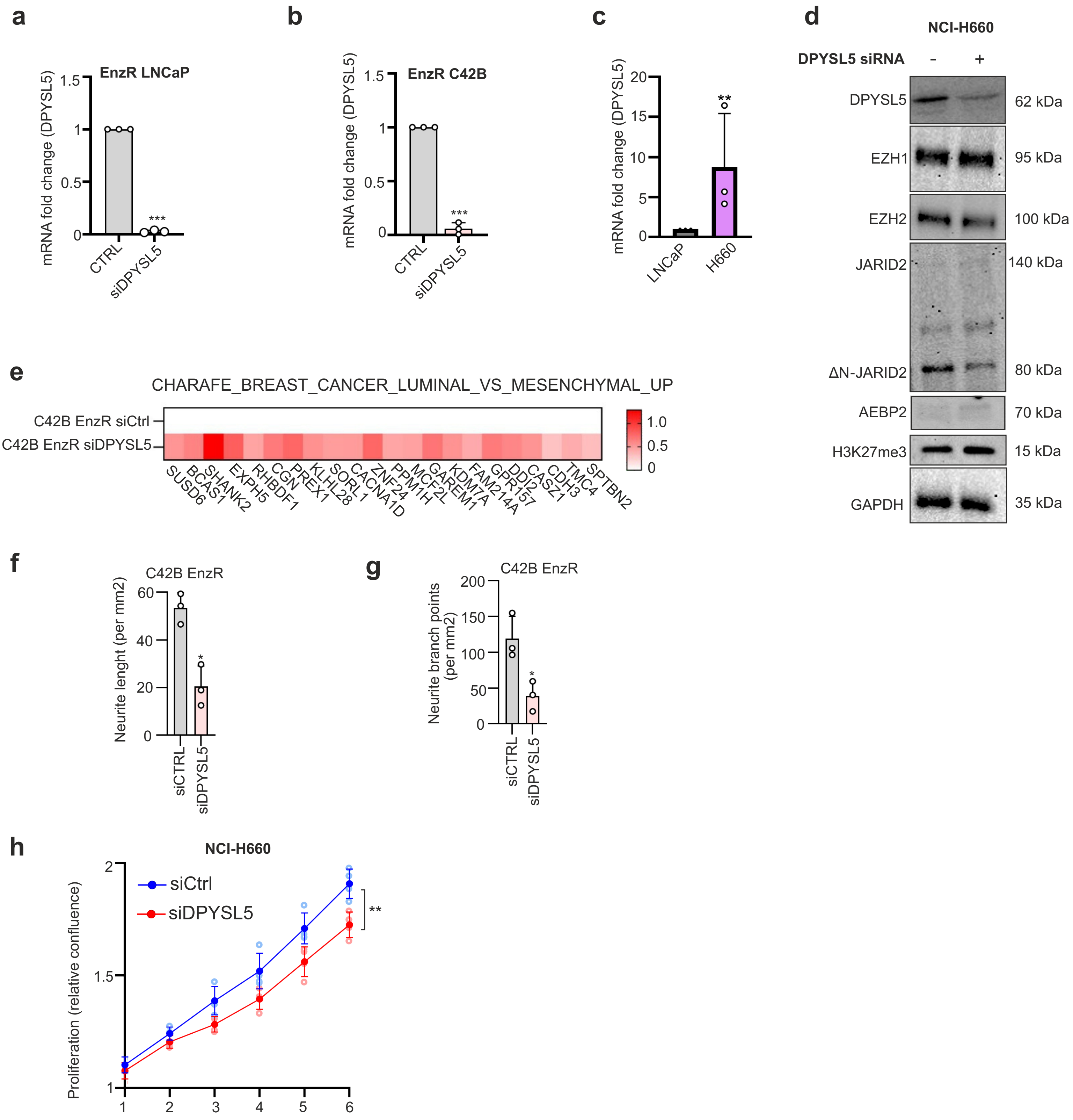

Supplementary Figure 4. Validation of DPYSL5 siRNA efficiency in a) LNCaP EnzR cells and in b) C42B EnzR cells. c) Expression of DPYSL5 is 7-folds higher in NCI-H660 cells than in LNCaP cells. d) Silencing DPYSL5 with siRNA in NCI-H660 cells lowers protein levels of truncated JARID2 and EZH2. e) DPYSL5 silencing in EnzR C42B cells upregulates luminal markers from CHARAFE_BREAST_CANCER_LUMINAL_VS_MESENCHYMAL_UP. f) Effect of DPYSL5 silencing in C42B-EnzR cells on neurite length and g) branch points. h) Silencing DPYSL5 with siRNA lowers proliferation in NCI-H660 cells when compared to control siRNA cells.

Figure 2g

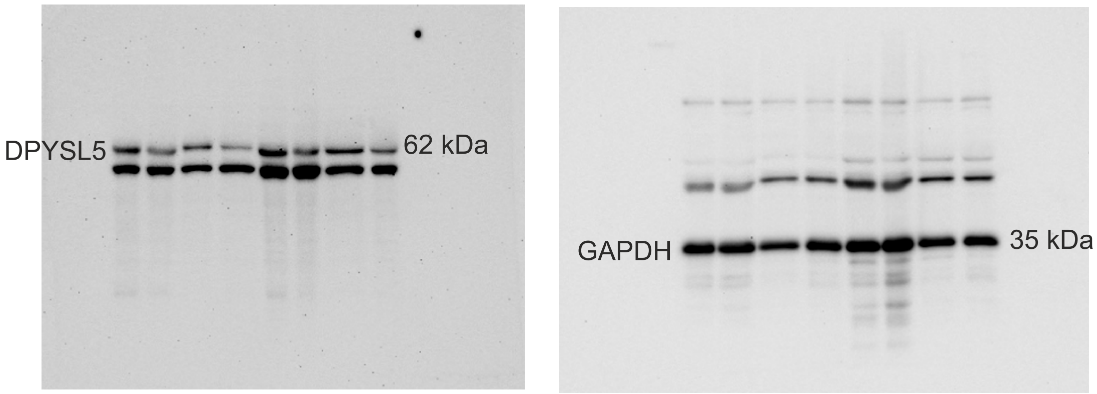

Figure 3e

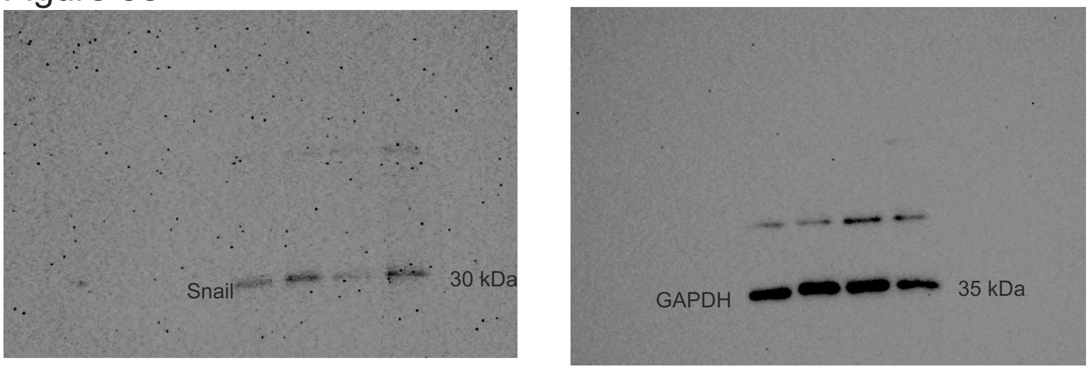

Figure 3j

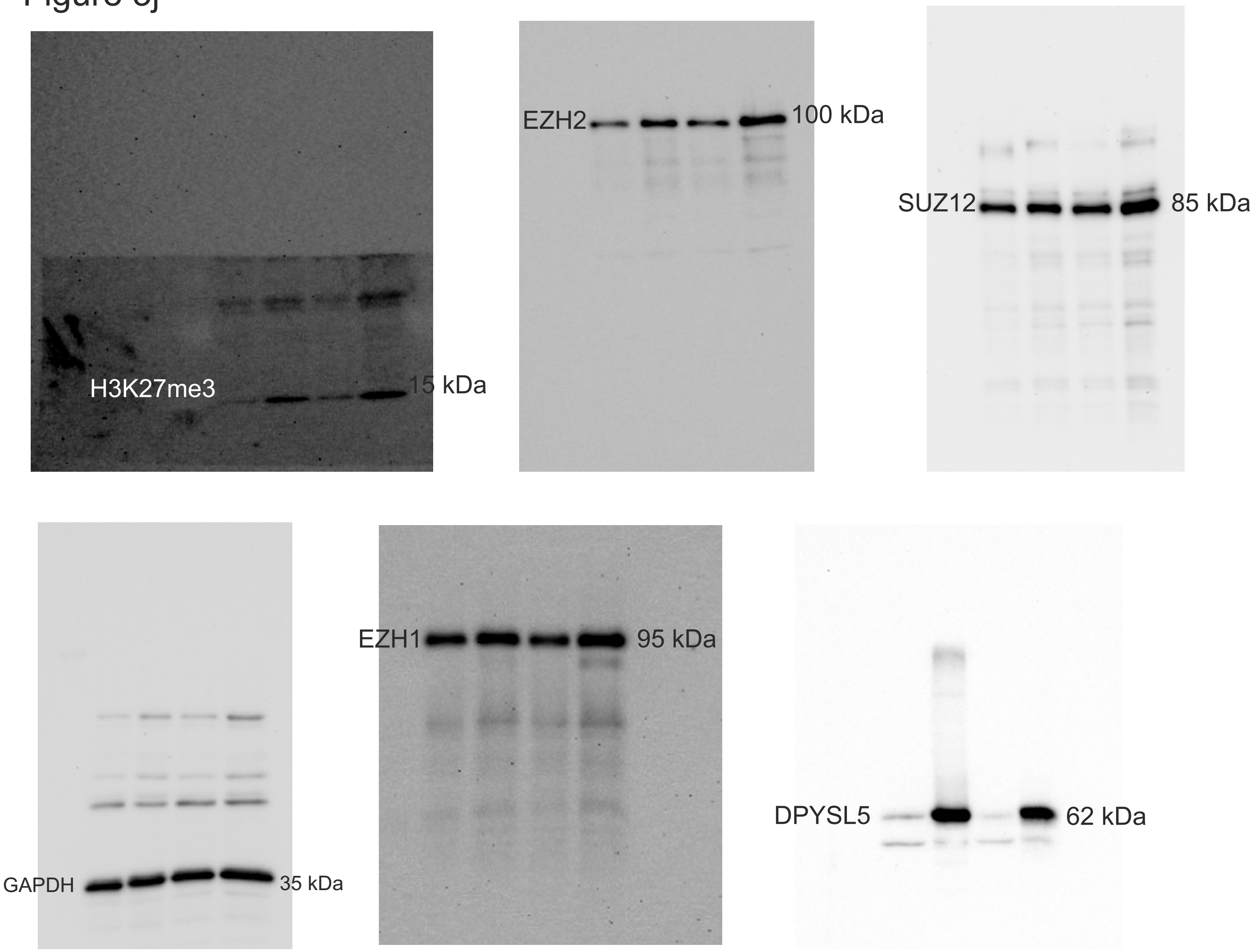

Supplementary Figure 5. Full membrane pictures of figures 2g, 3e and 3j

Figure 4b: LNCaP EnzR

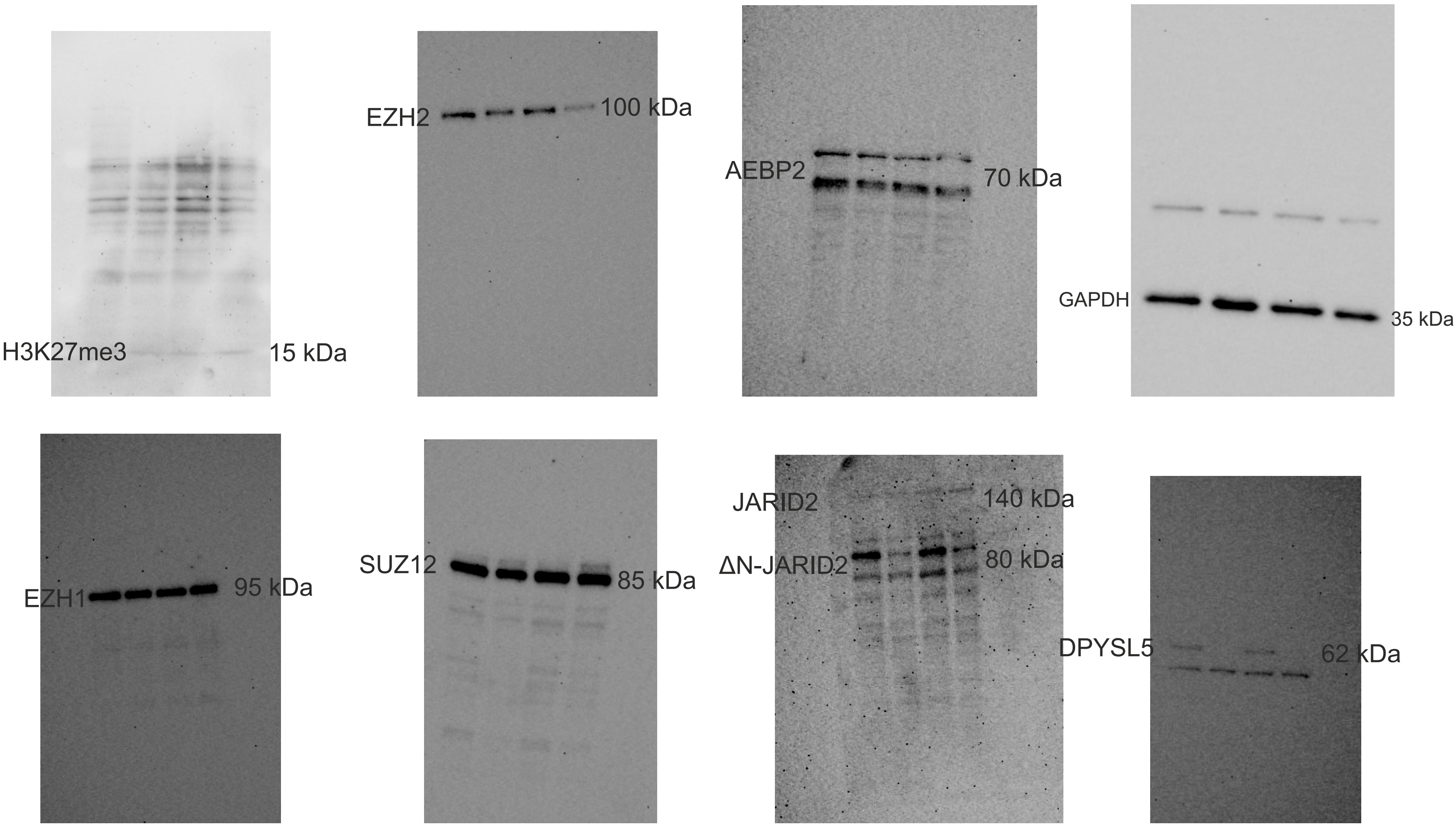

Figure 4b: C42B EnzR

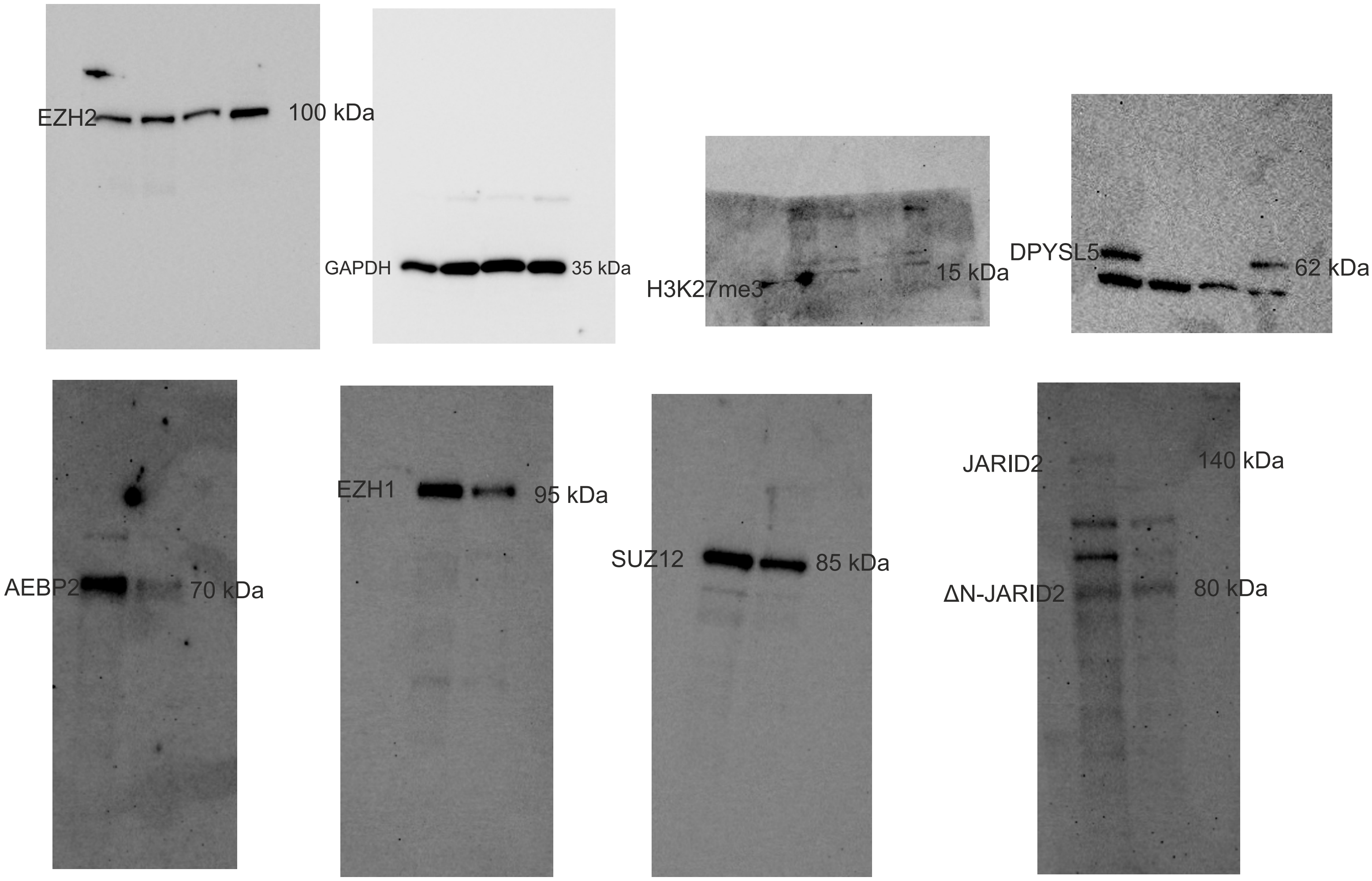

Supplementary Figure 6. Full membrane pictures of figure 4b

Supplementary Figure 4d: NCI-H660

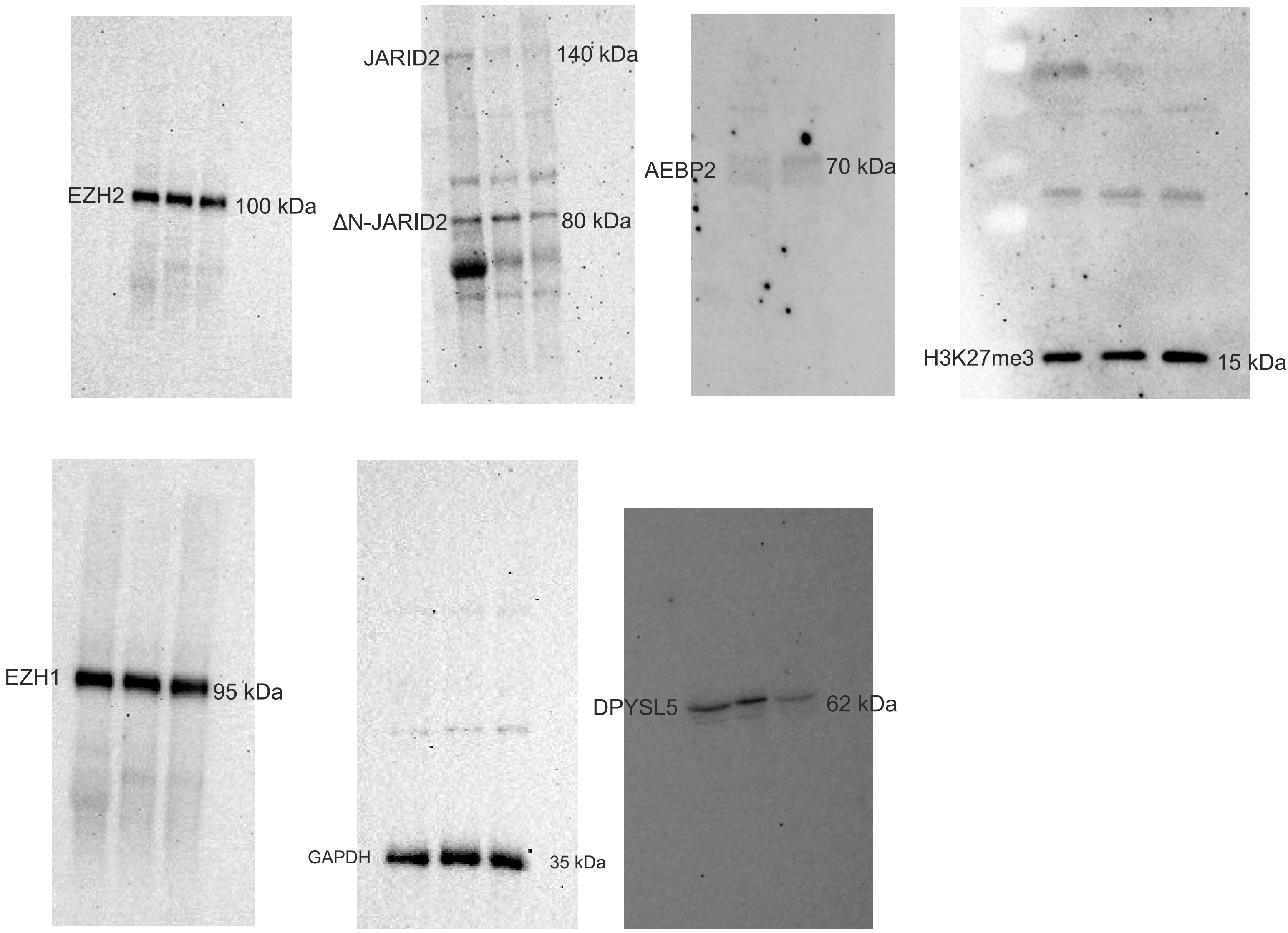

Supplementary Figure 7. Full membrane pictures of Supplementary Figure 4d

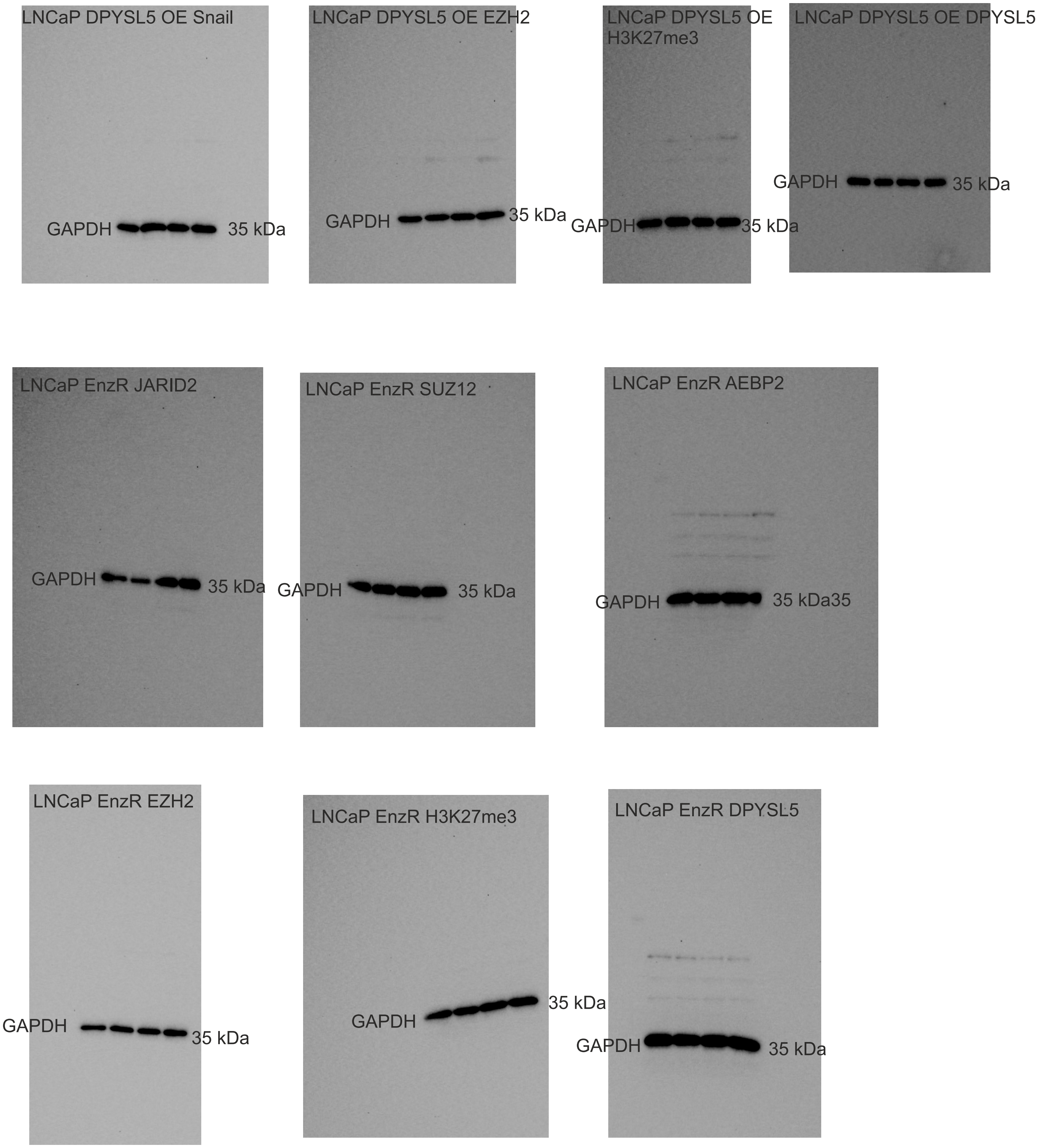

Supplementary Figure 8. Full membrane pictures of loading controls

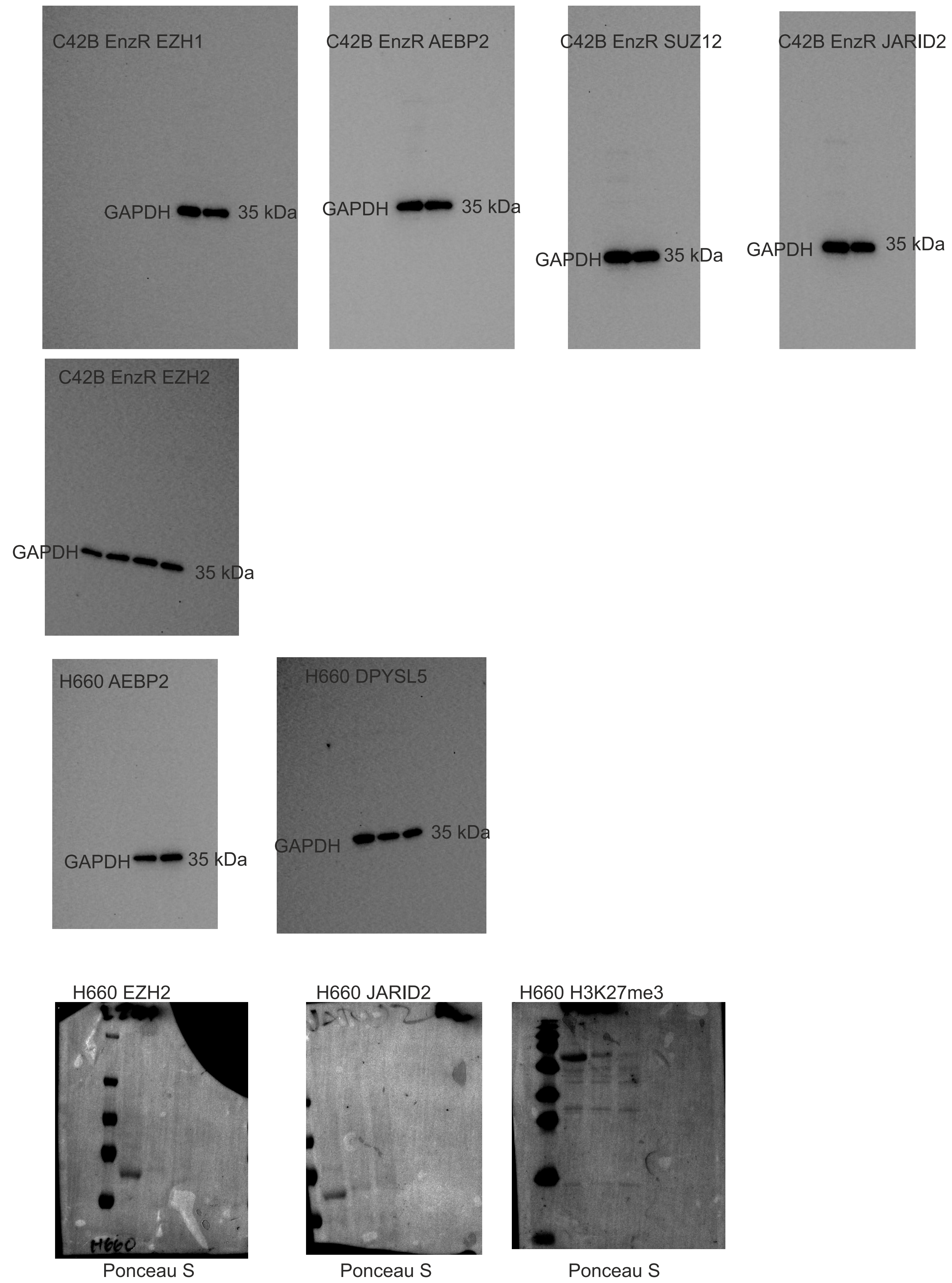

Supplementary Figure 9. Full membrane pictures of loading controls

Supplementary Table 1: Used primers

DPYSL5 Forward	GCTAAGGAGGCACTGGATTT
DPYSL5 Reverse	CTGGCTGAGATGGTCTTTGT
Chip DPYSL5 Forward	CAGTCCATTCTGGGGTCCATT
Chip DPYSL5 Reverse	GACTCCTCCAAACAGGGACAG
NANOG Forward	AGTCCCAAAGGCAAACAACCCACTTC
NANOG Reverse	TGCTGGAGGCTGAGGTATTTCTGTCTC
PSA Forward	CAC CCG AGC AGG TGC TTT TGC
PSA Reverse	GGC AGG TGC TTG TGG CCT CTC
SOX2 Forward	TGCGAGCGCTGCACA
SOX2 Reverse	TCATGAGCGTCTTGGTTTTCC
SYP Forward	TTAGTTGGGGACTACTCCTCG
SYP Reverse	GGCCCTTTGTTATTCTCTCGGTA
ASCL1 Forward	ACTTCACCAACTGGTTCTGAGTTTT
ASCL1 Reverse	TCAGAACCAGTTGGTGAAGTCGGTG
NSE Forward	GAACTATCCTGTGGTCTCC
NSE Reverse	CGACATTGGCTGTGAACTTG
CGA Forward	TCCAAGGCGCCAAGGA
CGA Reverse	CATCTTCAAAACCGCTGTGTTTC
GAPDH Forward	CATGAGAAGTATGACAACAGCT
GAPDH Reverse	AGTCCTTCCACGATACCAAAGT
