# Supplementary material for: DPYSL5 is highly expressed in treatment-induced neuroendocrine prostate cancer and promotes lineage plasticity via EZH2/PRC2
Source: Commun Biol. 2024 Jan 18;7:108. doi: 10.1038/s42003-023-05741-x (PMC10796342; doi:10.1038/s42003-023-05741-x)
Supplement: Supplementary file 2 — Description of Additional Supplementary Files [file 42003_2023_5741_MOESM2_ESM.docx]

**Description of Additional Supplementary Files**

**Supplementary File name:** Supplementary Data 1
**Description:** Source data and p-values for graphs
