# Supplementary material for: DPYSL5 is highly expressed in treatment-induced neuroendocrine prostate cancer and promotes lineage plasticity via EZH2/PRC2
Source: Commun Biol. 2024 Jan 18;7:108. doi: 10.1038/s42003-023-05741-x (PMC10796342; doi:10.1038/s42003-023-05741-x)
Supplement: Supplementary file 4 — Reporting Summary [file 42003_2023_5741_MOESM4_ESM.pdf]

nature portfolio

Corresponding author(s): Kirsi Ketola

Last updated by author(s): Nov 16, 2023

Reporting Summary

Nature Portfolio wishes to improve the reproducibility of the work that we publish. This form provides structure for consistency and transparency in reporting. For further information on Nature Portfolio policies, see our Editorial Policies and the Editorial Policy Checklist.

Statistics

For all statistical analyses, confirm that the following items are present in the figure legend, table legend, main text, or Methods section.

n/a	Confirmed	
☐	☒	The exact sample size (*n*) for each experimental group/condition, given as a discrete number and unit of measurement
☒	☐	A statement on whether measurements were taken from distinct samples or whether the same sample was measured repeatedly
☐	☒	The statistical test(s) used AND whether they are one- or two-sided<br>*Only common tests should be described solely by name; describe more complex techniques in the Methods section.*
☒	☐	A description of all covariates tested
☒	☐	A description of any assumptions or corrections, such as tests of normality and adjustment for multiple comparisons
☐	☒	A full description of the statistical parameters including central tendency (e.g. means) or other basic estimates (e.g. regression coefficient) AND variation (e.g. standard deviation) or associated estimates of uncertainty (e.g. confidence intervals)
☒	☐	For null hypothesis testing, the test statistic (e.g. *F*, *t*, *r*) with confidence intervals, effect sizes, degrees of freedom and *P* value noted<br>*Give P values as exact values whenever suitable.*
☒	☐	For Bayesian analysis, information on the choice of priors and Markov chain Monte Carlo settings
☒	☐	For hierarchical and complex designs, identification of the appropriate level for tests and full reporting of outcomes
☒	☐	Estimates of effect sizes (e.g. Cohen's *d*, Pearson's *r*), indicating how they were calculated

Our web collection on statistics for biologists contains articles on many of the points above.

Software and code

Policy information about availability of computer code

Data collection	RNA-seq libraries were prepared using NEBNext Poly(A) mRNA Magnetic Isolation Module (New England BioLabs, E7490) and NEBNext Ultra II Directional RNA Library Prep with Sample Purification Beads Kit (New England BioLabs, E7765). Pooled libraries were sequenced with NextSeq 500 at The EMBL Genomics Core Facility (Heidelberg, Germany).
Data analysis	Sequenced raw reads were quality controlled, the differential transcription was analyzed and differentially expressed gene sets were subjected to gene set enrichment analysis with GSEA.

For manuscripts utilizing custom algorithms or software that are central to the research but not yet described in published literature, software must be made available to editors and reviewers. We strongly encourage code deposition in a community repository (e.g. GitHub). See the Nature Portfolio guidelines for submitting code & software for further information.

Data

Policy information about availability of data

All manuscripts must include a data availability statement. This statement should provide the following information, where applicable:

- Accession codes, unique identifiers, or web links for publicly available datasets
- A description of any restrictions on data availability
- For clinical datasets or third party data, please ensure that the statement adheres to our policy

Generated RNA-seq datasets have been submitted to the NCBI Gene Expression Omnibus database (http://www.ncbi.nlm.nih.gov/geo/); accession code: GSE151433. Reviewer access token to GSE151433: orgrqaaynfcphin.

Research involving human participants, their data, or biological material

Policy information about studies with human participants or human data. See also policy information about sex, gender (identity/presentation), and sexual orientation and race, ethnicity and racism.

Reporting on sex and gender	The study describes the molecular mechanisms of male cancer, prostate cancer.
Reporting on race, ethnicity, or other socially relevant groupings	The study does not include clinical trial data including race, ethnicity or other socially relevant grouping.
Population characteristics	The study does not include clinical trial data describing population characteristics.
Recruitment	The study does not include clinical trial data recruitment.
Ethics oversight	Vancouver Prostate Centre, Department of Pathology at Vancouver General Hospital (Vancouver, Canada)

Note that full information on the approval of the study protocol must also be provided in the manuscript.

Field-specific reporting

Please select the one below that is the best fit for your research. If you are not sure, read the appropriate sections before making your selection.

☒ Life sciences ☐ Behavioural & social sciences ☐ Ecological, evolutionary & environmental sciences

For a reference copy of the document with all sections, see nature.com/documents/nr-reporting-summary-flat.pdf

Life sciences study design

All studies must disclose on these points even when the disclosure is negative.

Sample size	Sample size was determined by calculating population size for each experiment with confidence level of 95% and margin of error of 5%.
Data exclusions	No data was excluded from the analyses.
Replication	All attempts at repication were successful.
Randomization	The samples were randomly allocated into experimental groups.
Blinding	Blinding was not relevant for the study.

Reporting for specific materials, systems and methods

We require information from authors about some types of materials, experimental systems and methods used in many studies. Here, indicate whether each material, system or method listed is relevant to your study. If you are not sure if a list item applies to your research, read the appropriate section before selecting a response.

Materials & experimental systems

n/a	Involved in the study	
☐	☒	Antibodies
☐	☒	Eukaryotic cell lines
☒	☐	Palaeontology and archaeology
☒	☐	Animals and other organisms
☐	☒	Clinical data
☒	☐	Dual use research of concern
☒	☐	Plants

Methods

n/a	Involved in the study	
☒	☐	ChIP-seq
☒	☐	Flow cytometry
☒	☐	MRI-based neuroimaging

Antibodies

Antibodies used

Primary antibodies of DPYSL5 (Thermo Scientific Pierce CRMP5 (CCR-31) rat monoclonal US)
and GAPDH (Abcam anti-GAPDH (ab8245) mouse monoclonal, HRP-conjugated secondary antibodies (GE Healthcare ECL Anti-Rat IgG, HRP-linked whole ab and GE Healthcare ECL Anti-Mouse IgG, HRP-linked whole ab).

Validation

Antibodies were validated using western blot.

Eukaryotic cell lines

Policy information about cell lines and Sex and Gender in Research

Cell line source(s)

ATCC (all male)

Authentication

LNCaP and 22Rv1 cell lines were authenticated using Promega STR (Short Tandem Repeat) systems referenced to ATCC STR database by the Johns Hopkins Genetic Resources Core Facility (JHGRCF). PC-3 cell line was authenticated at Genomics Unit of Technology Centre in Institute for Molecular Medicine Finland (FIMM) using the Promega GenePrint24 System.

Mycoplasma contamination

All cells were tested negative for mycoplasma contamination

Commonly misidentified lines
(See ICLAC register)

Name any commonly misidentified cell lines used in the study and provide a rationale for their use.

Clinical data

Policy information about clinical studies

All manuscripts should comply with the ICMJE guidelines for publication of clinical research and a completed CONSORT checklist must be included with all submissions.

Clinical trial registration

Not applicable. The clinical data were not extracted from a clinical trial.

Study protocol

This is a retrospective analysis on clinically annotated biospecimens banked in the VPC institutional biobank.

Data collection

Patients with localized and metastatic prostate cancer treated at the Vancouver Prostate Centre were consented for archival tissue retrieval and analysis, from Sep 2009 to Oct 2022, REB: H09-01628. For this specific analysis, treatment-naive prostate adenocarcinoma, CRPC and neuroendocrine prostate cancer tissues were included.

Outcomes

For this analysis, immunoistochemistry was used on the prostate cancer specimens. No clinical outcomes correlative studies were performed.
